# Epicardial placement of human MSC-loaded fibrin sealant films for heart failure: Preclinical efficacy and mechanistic data

**DOI:** 10.1016/j.ymthe.2023.12.016

**Published:** 2023-12-22

**Authors:** Laura Fields, Tomoya Ito, Kazuya Kobayashi, Yuki Ichihara, Mihai-Nicolae Podaru, Mohsin Hussain, Kizuku Yamashita, Vanessa Machado, Fiona Lewis-McDougall, Ken Suzuki

## Main text

(Molecular Therapy *29*, 2554–2570; August 2021)

In the originally published version of this article, there were errors in the cell culture images of Figure S8A. The corrected version is shown below. This correction does not alter the graph, result, or conclusion of the study. The authors would like to apologize for any inconvenience caused.

**Figure:**
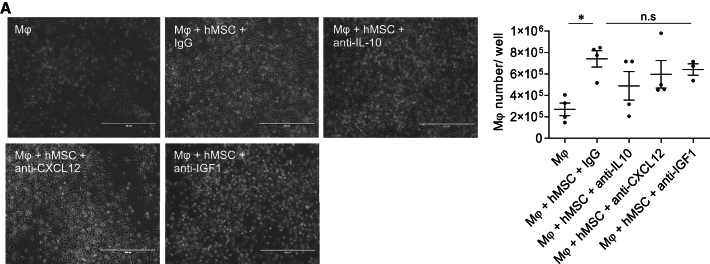
Figure S8. Inhibition of hAM-MSC-secreted PGE2, IL-10, CXCL12 or IGF1 did not affect Mφ proliferation (corrected) (A) Mouse bone marrow-derived Mφ were co-cultured for 48 with hAM-MSCs in the presence of neutralizing antibodies for human IL-10 (Mφ + hMSC + anti-IL-10), IGF1 (Mφ + hMSC + anti-IGF1), CXCL12 (Mφ + hMSC + anti-CXCL12) or IgG antibody controls (Mφ + hMSC + IgG). Cells were collected and the numbers were quantified. Representative phase contrast images and a chart showing the cell number counted are presented. This experiment was carried out in conjunction with the Figure 7C study. The same images and data of the control groups (Mφ group and Mφ + hMSC + IgG group) are presented in Figure 7 and Figure S8. Scale bar = 400 µm. n = 3–4 per group.

