# Supplementary material for: Epicardial placement of human MSC-loaded fibrin sealant films for heart failure: Preclinical efficacy and mechanistic data
Source: Mol Ther. 2021 Apr 20;29(8):2554–70. doi: 10.1016/j.ymthe.2021.04.018 (PMC8353205; doi:10.1016/j.ymthe.2021.04.018)
Supplement: Document S1. Figures S1–S8 and Table S1 [file mmc1.pdf]

YMTHE, Volume *29*

Supplemental Information

Epicardial placement of human MSC-loaded fibrin sealant films for heart failure: Preclinical efficacy and mechanistic data

Laura Fields, Tomoya Ito, Kazuya Kobayashi, Yuki Ichihara, Mihai-Nicolae Podaru, Mohsin Hussain, Kizuku Yamashita, Vanessa Machado, Fiona Lewis-McDougall, and Ken Suzuki

SUPPLEMENTAL INFORMATION

Epicardial Placement of Fibrin Sealant Film-incorporating Human Mesenchymal Stromal Cells for the Treatment of Heart Failure: Towards Clinical Translation and Mechanistic Implication

Short Title: Fields et al. MSC-Dressing Therapy

Supplemental Information includes:

- Supplemental Figures S1-S8 with legends
- Supplemental Table S1

Figure S1

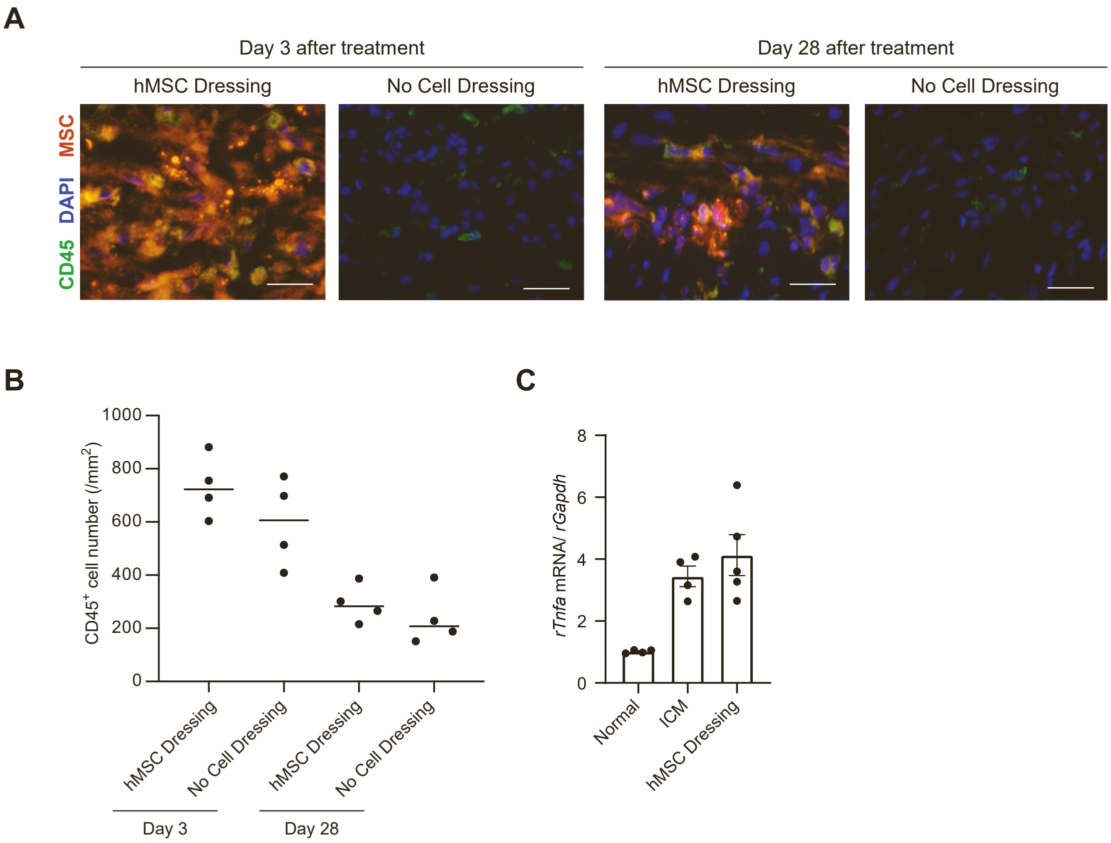

Figure S1. Immunological response induced by hAM-MSC-dressing therapy.

Four weeks after left coronary artery ligation in rats, a hAM-MSC-dressing (a fibrin sealant film containing 2×10^6 hAM-MSCs; hMSC-dressing group), a fibrin sealant film only (No Cell-dressing group), or nothing (no treatment; ICM group) were placed onto the rat heart surface.

(A) Representative images of immunohistolabeling for CD45 (green) with (DAPI; blue) staining exhibiting the CD45$^+$ cell accumulation in the construct of hAM-MSC-dressing or No Cell-dressing at day 3 and 28 after treatment. Scale bars = 20 µm. n = 4 rats in each group.

(B) The graphs presenting the number of CD45$^+$ cells in the construct of hAM-MSC-dressing or No Cell-dressing at day 3 and 28 after treatment. n = 4 rats in each group.

(C) RT-qPCR analysis data showing increased expression of *rTnfa* genes of the rat LV samples of the hAM-MSC group, as compared to the ICM group, at day 3 post treatment. Normal (no MI, no treatment) group was included as a reference. n = 5 hearts in each group. Data are presented as mean ± SEM, Student's t-test for statistical analysis of (B); One-way ANOVA with Tukey's post-hoc tests for (C).

Figure S2

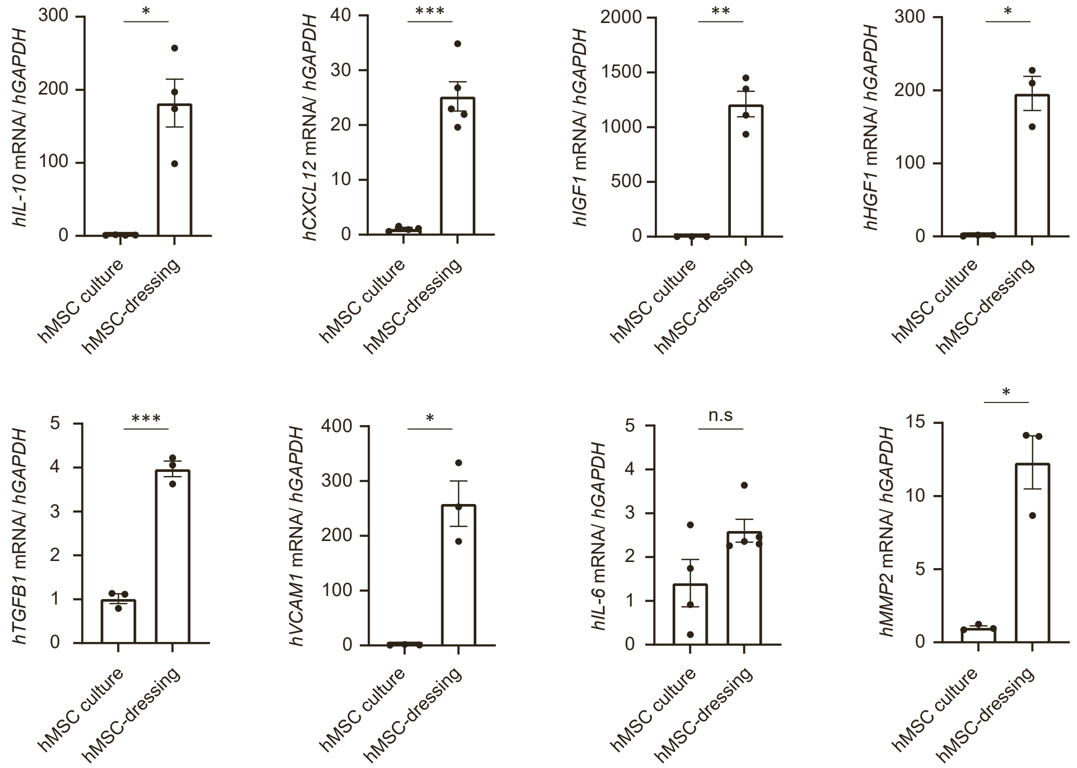

Figure S2. Expression of human reparative genes in the rat heart post hAM-MSC-dressing therapy.

Four weeks after left coronary artery ligation in rats, hAM-MSC-dressings (fibrin sealant film containing 2×10^6 hAM-MSCs; hMSC-dressing group) were placed onto the heart surface. At day 3 post-transplantation, RNA was extracted from the rat LV tissue for RT-qPCR for tissue repair-related human genes. As a control, RNA was extracted from ordinarily cultured hAM-MSCs was used. n = 5 in each group. Data are presented as mean ± SEM, $*p < 0.05$, $**p < 0.01$, $***p < 0.001$, n.s = not significant. Student's t-test.

Figure S3

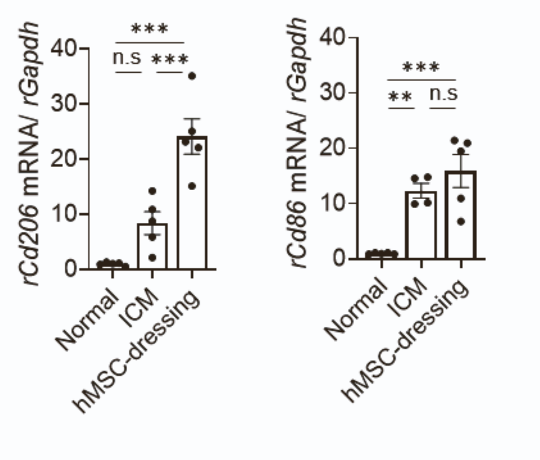

Figure S3. Expression of Mφ phenotype markers in the rat heart post hAM-MSC-dressing therapy.

Rat hearts were collected at Day 3 after hAM-MSC-dressing therapy or no treatment (ICM group) in a rat ICM model and subjected to RT-qPCR for *Cd206* and *Cd86*. Normal (no MI, no treatment) group was included as a reference. n = 5 hearts in each group. Data are presented as mean ± SEM, **p < 0.01, ***p < 0.001, n.s = not significant. One-way ANOVA with Tukey's post-hoc test.

Figure S4

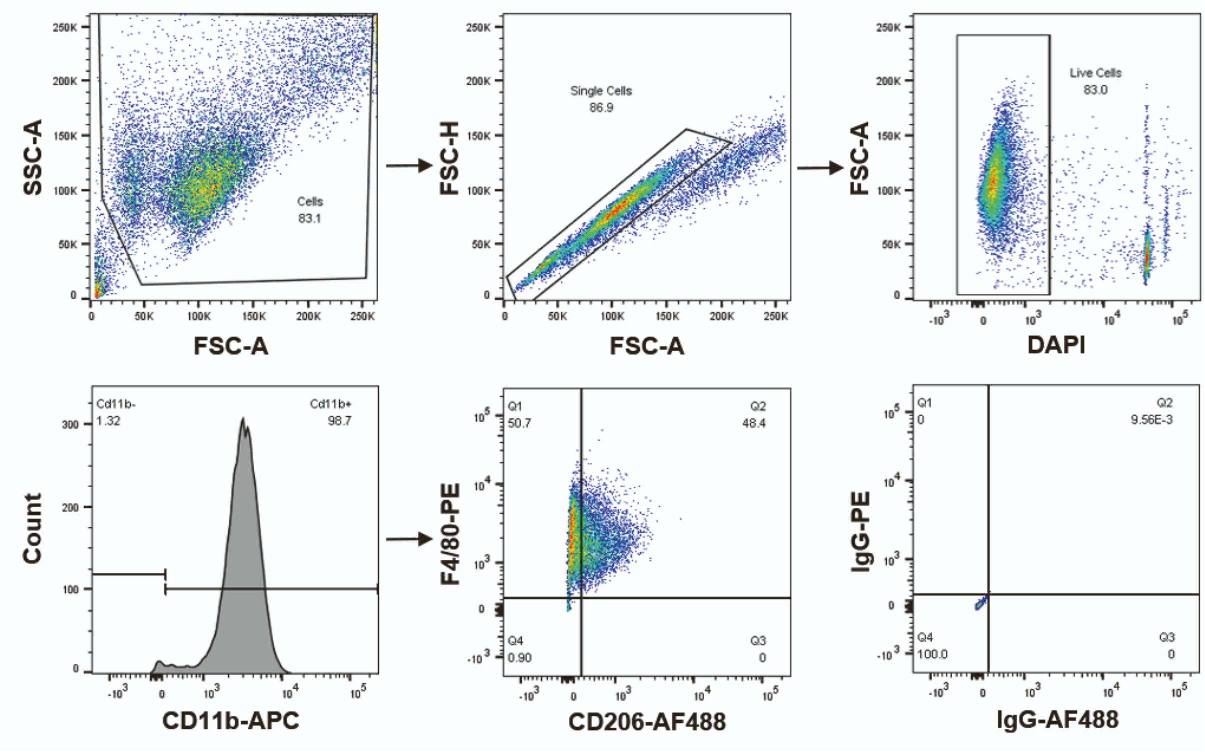

Figure S4. Flow cytometry gating to characterize M2Mφ.

Gating strategy to detect M2Mφ by flow cytometry is illustrated. Debris, doublets and dead cells are excluded. Cells double positive for F4/80 and CD206 within the $CD11b^+$ population were defined as M2Mφ by flow cytometry. Representative dot plots from a sample of M-CSF + hAM-MSC treated mouse BM-MNCs are presented. The same cells stained with IgG conjugated fluorescent antibodies shown as negative control. FMO control also used.

Figure S5

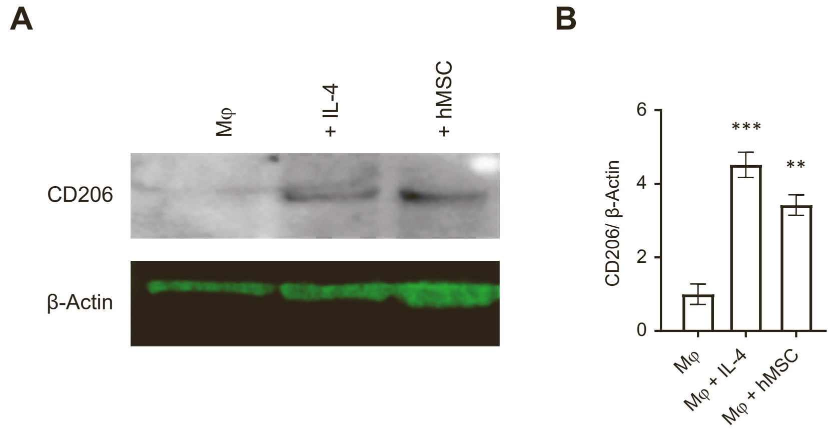

Figure S5. Enhanced CD206 protein expression in Mφ by coculture with hAM-MSCs.

Mouse bone marrow-derived Mφ were cultured alone, with IL-4 (20ng/ ml) or hAM-MSCs in a non-contact co-culture model for 48 hours. Cells were collected and CD206 protein expression levels were analyzed by western blot technique.

(A) Representative blot showing higher CD206 expression in Mφ after IL-4 and hAM-MSC treatment compared to unstimulated Mφ. β-Actin was used as loading control.

(B) Quantification of CD206 expression. n = 3 animals. Data are presented as mean fold change ± SEM compared to Mφ, $**p < 0.01$, $***p < 0.001$, One-way ANOVA with Dunnett's post-hoc test.

Figure S6

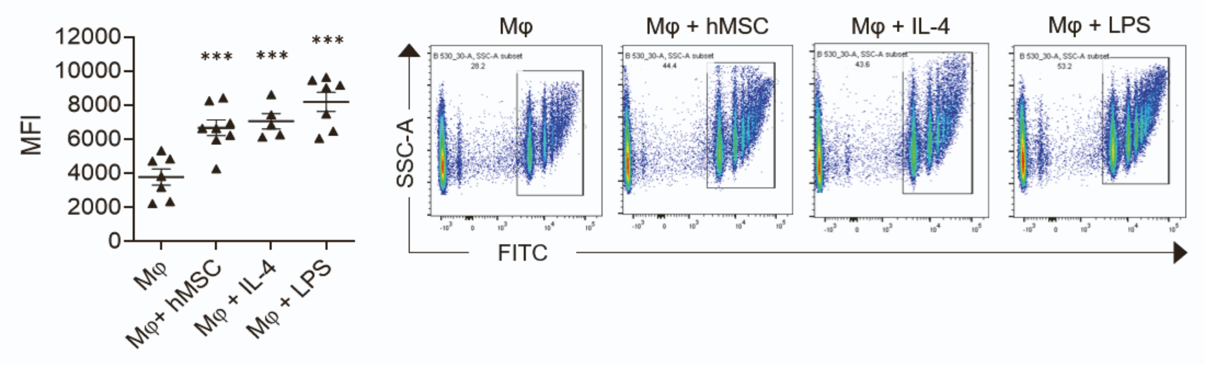

Figure S6. Increased Mφ phagocytotic activity by co-culture with hAM-MSCs.

Mouse bone marrow-derived Mφ were subjected to hAM-MSC transwell co-culture conditions before adding FITC-labelled beads in serum free media. Positive controls of LPS (50ng/ ml) or IL-4 (20ng/ ml) administration was included as well as an unstimulated (MCSF only) Mφ treatment group. Cells containing the $FITC^{+}$ beads were assessed by flow cytometry. The mean fluorescence intensity (MFI) was quantified (left graph). Representative flow cytometry images for each group are shown in the panel on the right. n = 5-7. Data are presented as mean ± SEM, ***$p < 0.001$ compared to Mφ, One-way ANOVA with Dunnett's post-hoc test.

Figure S7

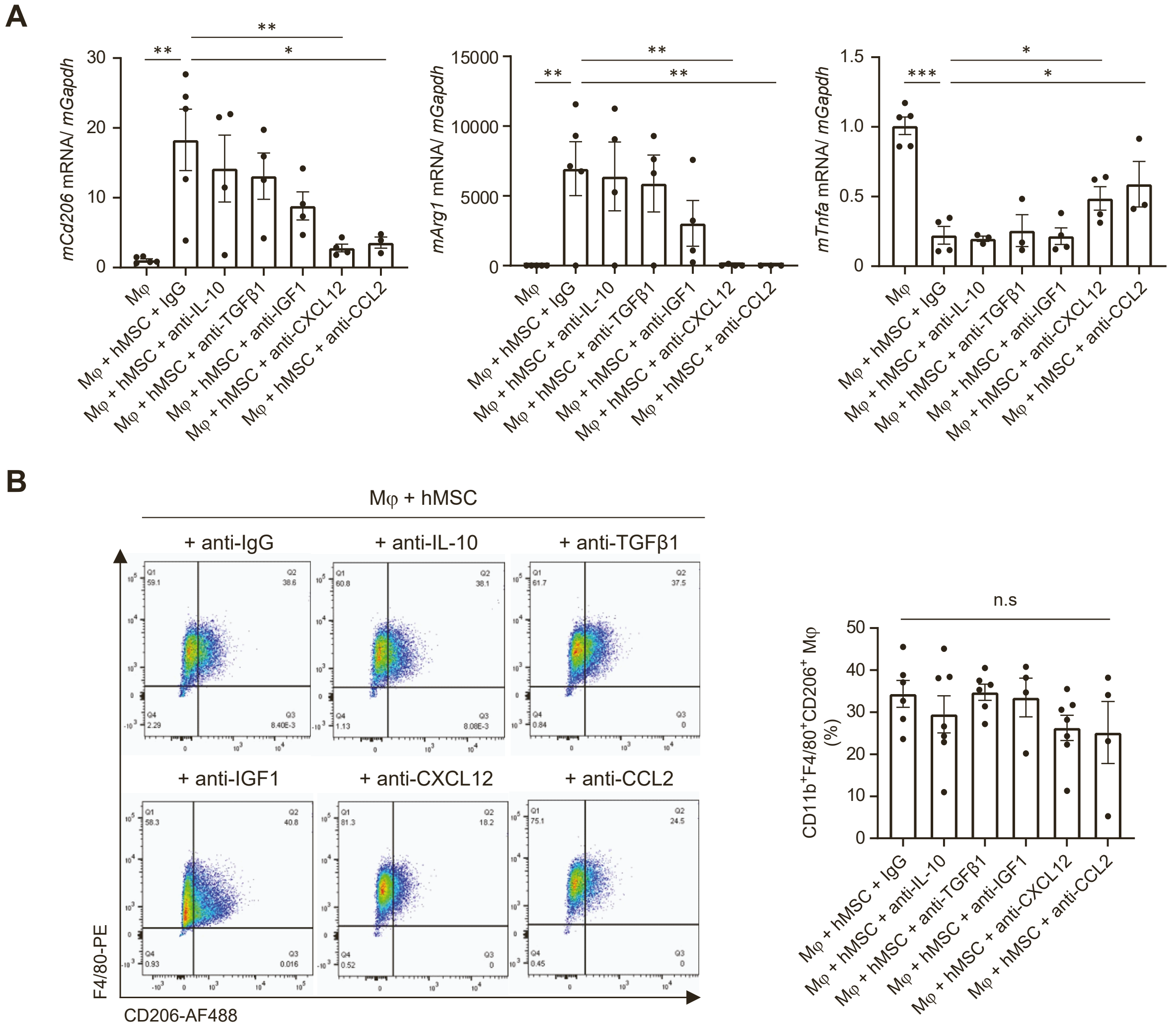

Figure S7. Role of CXCL12 and CCL2 in hAM-MSC-induced M2Mφ polarization.

Mouse bone marrow-derived Mφ were mono-cultured (Mφ group) or co-cultured with hAM-MSCs in the presence of neutralizing antibodies for human IL-10 (Mφ + hMSC + anti-IL-10), TGFβ1 (Mφ + hMSC + anti-TGFβ1), IGF1 (Mφ + hMSC + anti-IGF1), CXCL12 (Mφ + hMSC + anti-CXCL12) and CCL2 (Mφ + hMSC + anti-CCL2) or IgG antibody control (Mφ + hMSC + IgG). After 48 hours Mφ were collected and assessed for M2Mφ production.

(A) Gene expression of Mφ in each group measured by RT-qPCR. *Gapdh* was used as a reference gene. n = 4-5 biological replicates with 3 technical replicates.

(B) The percentage of M2Mφ ($CD11b^+F4/80^+CD206^+$) to the total live cell population quantified by flow cytometry. Representative dot plots and a bar chart summarizing the data are presented. n = 4-8 in each group.

Data are presented as mean ± SEM. *$p < 0.05$, **$p < 0.01$, ***$p < 0.001$. One-way ANOVA with Dunnett's multiple comparisons test.

Figure S8

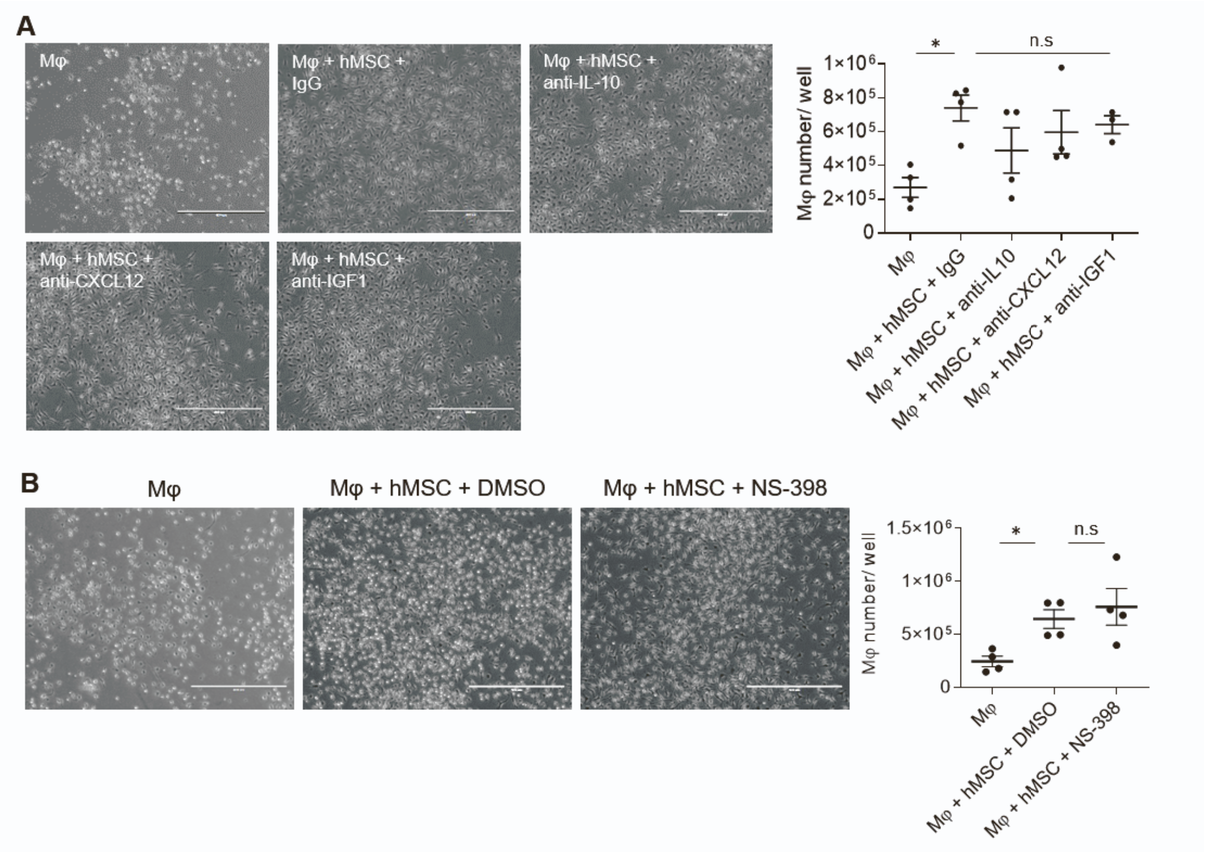

Figure S8. Inhibition of hAM-MSC-secreted PGE2, IL-10, CXCL12 or IGF1 did not affect Mφ proliferation.

(A) Mouse bone marrow-derived Mφ were co-cultured for 48 with hAM-MSCs in the presence of neutralizing antibodies for human IL-10 (Mφ + hMSC + anti-IL-10), IGF1 (Mφ + hMSC + anti-IGF1), CXCL12 (Mφ + hMSC + anti-CXCL12) or IgG antibody controls (Mφ + hMSC + IgG). Cells were collected and the numbers were quantified. Representative phase contrast images and a chart showing the cell number counted are presented. Scale bar = 400 µm. n=3-4 per group.

(B) Mouse bone marrow-derived Mφ were co-cultured for 48 with hAM-MSCs pre-treated with DMSO (vehicle control; Mφ + hMSC + DMSO group) or COX-2 inhibitor, NS-398, in DEMSO (Mφ + hMSC + NS-398 group). Representative phase contrast images and a chart showing the cell number counted are presented. Scale bar = 400 µm. n = 4 in each group.

Data are presented as mean ± SEM. $*p < 0.05$; n.s = not significant. One-way ANOVA with Dunnett's comparisons test.

Table S1. Primer sequences

Gene	Primer Sequence
mArg1	Forward:5'-CAAGACAGGGCTCCTTTCAG-3'<br>Reverse: 5'-AAGCAAGCCAAGGTTAAAGC-3'
mFizz1	Forward:5'-AGGAACTTCTTGCCAATCCA-3'<br>Reverse: 5'-ACAAGCACACCCAGTAGCAG-3'
mIl-10	Forward:5'-GGACAACATACTGCTAAGGACTCCT-3'<br>Reverse: 5'-GCCTGGGGCATCACTTCTAC-3'
mTgfb1	Forward:5'-CCTATATTTGGAGCCTGGACACAC-3'<br>Reverse: 5'-GCTTGCGACCCACGTAGTAGA-3'
mIgf1	Forward:5'-GGACCGAGGGGCTTTTACTTC-3'<br>Reverse: 5'-GGCACAGTACATCTCCAGTCTCCTC-3'
mTnfa	Forward:5'-ATGGCCTCCCTCTGATCAGTT-3'<br>Reverse: 5'-TCTTTGAGATCCATGCCGTTG-3'
mCd206	Forward:5'-ACTACACACTCATCCATTACAACCAA-3'<br>Reverse: 5'-GGCACCTATCACAATCAGGAGGA-3'
mInos	Forward:5'- CTCCATGACTCCCAGCACAAA-3'<br>Reverse: 5'- CACTCTCTTGCGGACCATCTC-3'
mIl-1b	Forward:5'- CAAGCAACGACAAAATACCTGTG-3'<br>Reverse: 5'- ACCGTTTTTCCATCTTCTTCTTTGG-3'
mCd86	Forward:5'- GTTACTGTGGCCCTCCTCCTTGT-3'<br>Reverse: 5'- TGTCAGCGTTACTATCCCGCTCT-3'
rCxcl12	Forward:5'- CTGGATAATGTGAGAACATGCCTAGAA-3'<br>Reverse: 5'- TGCAAAAGTCCAATTCCTCAAA-3'
rVcam1	Forward:5'- CTGAGTGCAAGAAGCCAACTAGAAA-3'<br>Reverse: 5'- CTACTACTCTAAACGACCTCGCAATGA-3'
rIl-10	Forward:5'- CACCCGGCATCTACTGGACT-3'<br>Reverse: 5'- TATTTTGGAGAGAGGTACAAACGAGGT-3'
rIl-6	Forward:5'- GCCTAAGCATATCAGTTTGTGGACATT-3'<br>Reverse: 5'- AACATTCATATTGCCAGTTCTTCGT-3'
rTgfb1	Forward:5'- GACTCTCCACCTGCAAGACCATC-3'<br>Reverse: 5'- GGACTGGCGAGCCTTAGTTTG-3'

rIl-4	Forward:5'- CTGTAGAGAGCTATTGATGGGTCTCAG-3'<br>Reverse: 5'- CTTTTTCTGTGACCTGGTTCAAAGTG-3'
rIgf1	Forward:5'- AAACAAATAGAATAACAATAACTATGACTTTGAGG-3'<br>Reverse: 5'- TTGAAGGAACCATTGAGAGTTTAAGAG-3'
hCXCL12	Forward:5'- CTTGTAATCCGAATCTCTTTTTGCTTT-3'<br>Reverse: 5'- GCCCAAGGGAGTGTCAGGTAG-3'
hVCAM1	Forward:5'- TACTGCTCATCATTCCTTGAGAAAAAC-3'<br>Reverse: 5'- GCTCACAGCAAGGGACATAGA-3'
hIL-10	Forward:5'- ACGCTTTCTAGCTGTTGAGCTGTTTT-3'<br>Reverse: 5'- GGCTCCCTGGTTTCTCTTCCT-3'
hIL-6	Forward:5'- ATGGGCACCTCAGATTGTTGTT-3'<br>Reverse: 5'- GTGTCCTAACGCTCATACTTTTAGTTC-3'
hTGFB1	Forward:5'- CTCATTCAGTCACCATAGCAACACTC-3'<br>Reverse: 5'- CTTCTTCACTATCCCCCACTAAAGCA-3'
hIL-4	Forward:5'- ACTGCTTCCCCCTCTGTTCT-3'<br>Reverse: 5'- CTTCTGCTCTGTGAGGCTGTT-3'
hIGF1	Forward:5'- ACCACCTTTCAACTTTTTATCACTCAC-3'<br>Reverse: 5'- CAACAAAACAATGGAGCCTTCTAAC-3'
