# Supplementary material for: Epicardial placement of human MSC-loaded fibrin sealant films for heart failure: Preclinical efficacy and mechanistic data
Source: Mol Ther. 2021 Apr 20;29(8):2554–70. doi: 10.1016/j.ymthe.2021.04.018 (PMC8353205; doi:10.1016/j.ymthe.2021.04.018)
Supplement: Document S2. Article plus supplemental information [file mmc2.pdf]

Molecular Therapy
Original Article

Epicardial placement of human MSC-loaded fibrin sealant films for heart failure: Preclinical efficacy and mechanistic data

Laura Fields,[1] Tomoya Ito,[1] Kazuya Kobayashi,[1] Yuki Ichihara,[1] Mihai-Nicolae Podaru,[1] Mohsin Hussain,[1] Kizuku Yamashita,[1] Vanessa Machado,[1] Fiona Lewis-McDougall,[1] and Ken Suzuki[1]

[1]William Harvey Research Institute, Barts and The London School of Medicine and Dentistry, Queen Mary University of London, London, UK

Mesenchymal stromal cell (MSC) transplantation has been investigated as an advanced treatment of heart failure; however, further improvement of the therapeutic efficacy and mechanistic understanding are needed. Our previous study has reported that epicardial placement of fibrin sealant films incorporating rat amniotic membrane-derived (AM)-MSCs (MSC-dressings) could address limitations of traditional transplantation methods. To progress this finding toward clinical translation, this current study aimed to examine the efficacy of MSC-dressings using human AM-MSCs (hAM-MSCs) and the underpinning mechanism for myocardial repair. Echocardiography demonstrated that cardiac function and structure were improved in a rat ischemic cardiomyopathy model after hAM-MSC-dressing therapy. hAM-MSCs survived well in the rat heart, enhanced myocardial expression of reparative genes, and attenuated adverse remodeling. Copy number analysis by qPCR revealed that upregulated reparative genes originated from endogenous rat cells rather than hAM-MSCs. These results suggest hAM-MSC-dressing therapy stimulates a secondary release of paracrine factors from endogenous cells improving myocardial repair ("secondary paracrine effect"), and cardiac M2-like macrophages were identified as a potential cell source of repair. We demonstrated hAM-MSCs increased M2-like macrophages through not only enhancing M2 polarization but also augmenting their proliferation and migration capabilities via PGE_2, CCL2, and TGF-β1, resulting in enhanced cardiac function after injury.

INTRODUCTION

Myocardial infarction (MI) and post-MI ischemic cardiomyopathy (ICM) is an increasing clinical, social, and financial burden.[1] A large number of pre-clinical studies have indicated that mesenchymal stromal cell (MSC) transplantation is a promising approach for the treatment of this fatal disease,[2,3] and clinical trials have established the safety and feasibility.[4,5] Although the clinical efficacy data observed to date are mixed, meta-analysis studies detected a positive therapeutic effect by MSC therapy.[6] Having said this, for future clinical successes of this approach, it is essential to augment the therapeutic efficacy, which could be achieved through refining the currently sub-optimal protocols including the choice of cell-delivery route and MSC quality. In addition, further investigations on mechanistic insights of MSC therapy are also important.

Current MSC delivery routes to the heart are intramyocardial, intracoronary, or intravenous injections; however, these methods result in poor donor cell engraftment, limiting the therapeutic efficacy.[7,8] We have recently developed an innovative technique: epicardial placement of bi-layered, fibrin sealant films incorporating MSCs (MSC-dressing).[9] This product can be easily and instantly fabricated using a commercially available fibrin sealant film and MSC suspension at the site of treatment. The MSC-dressing is formed of two layers. The inner layer consists of a fibrin-MSC complex that enables its prompt and firm adherence to the heart surface without sutures or extra adhesives and also enhances the reparative ability of incorporated MSCs. The outer collagen sponge protects the MSC-fibrin complex from abrasion by the surrounding tissues, improving donor cell retention and survival.[9] As an MSC type, an increasing number of reports propose the advantages of amniotic membrane-derived MSCs (AM-MSCs) in terms of mass production as well as immunomodulative and tissue-repair abilities.[2,10,11] We have previously reported that epicardial placement of a rat-derived AM-MSC-dressing markedly increased donor cell engraftment and enhanced cardiac function in a syngeneic rat ICM model compared to intramyocardial injection.[9] For further development of this AM-MSC-dressing therapy toward clinical application, we confirm that human (h)AM-MSC-dressings are feasible, safe, and functional in a rat ICM model. The previously reported observation that MSCs of this cell source survived well without causing significant immunological responses in an immunocompetent rat heart[10] would validate this human-to-rat xenogeneic cell-transplantation model for the purpose of characterizing hAM-MSC-dressings.

Received 2 November 2020; accepted 15 April 2021;
https://doi.org/10.1016/j.ymthe.2021.04.018.

Correspondence: Ken Suzuki, William Harvey Research Institute, Barts and The London School of Medicine and Dentistry, Queen Mary University of London, Charterhouse Square, London EC1M 6BQ, UK.
E-mail: ken.suzuki@qmul.ac.uk

 © 2021 The Author(s).
This is an open access article under the CC BY license (http://creativecommons.org/licenses/by/4.0/).

The mechanism by which transplanted MSCs improve cardiac function remains to be fully elucidated. As trans-differentiation of MSCs to cardiomyocytes rarely occurred *in vivo*, it is widely agreed that the MSC-derived therapeutic effects are mediated by their secretome, which induces improvement of microvascular formation, resolution of inflammation, reduction of interstitial pathological fibrosis, and attenuation of cardiomyocyte hypertrophy.[12,13] These are "direct" paracrine effects, which are achieved by the secretome from hAM-MSCs directly activating cardiac cells, i.e., cardiomyocytes, vascular cells, and/or cardiac fibroblasts. However, given the extremely poor donor cell survival post-transplantation, reparative secretion from donor cells may not be sufficient to fully explain the extensive myocardial repair achieved by MSC therapy. In this viewpoint, we and others have proposed an alternative mechanism, namely, the "secondary (or indirect)" paracrine effect, in which endogenous host cells are stimulated by hAM-MSC's secretome, and the triggered host cells, in turn, secrete myocardial repair-related factors, promoting cardiac tissue repair through activation of cardiac cells. However, further supporting data are needed to fully elucidate this concept of the secondary paracrine effect. To this end, we would take advantage of the xenogeneic model of hAM-MSC-dressing transplantation to the rat heart in this study. This model was anticipated to enable precise dissection of the origin of paracrine effect-related factors, either donor hAM-MSCs or host rat tissue, by using species specific identification, providing previously unidentified information to understand the direct versus secondary paracrine mechanisms.

As the central mediator cells of the secondary paracrine effect post-MSC transplantation, M2-like reparative macrophages (M2Mφ) have been suggested.[14–16] Shortly after MI, pro-inflammatory Mφ are recruited into the injured myocardium to remove necrotic cell debris.[17] Subsequently, 5−7 days after MI, M2Mφ become dominant and contribute to myocardial repair through their secretome. MSC transplantation is known to increase the post-MI accumulation of cardiac M2Mφ.[16,18,19] This change has been reported to be underpinned by MSC's secretome-induced polarization of Mφ to a M2-like phenotype, in which distinct proteome and/or exosome are likely to play a role.[19–24] However, the number of M2Mφ in the heart is regulated, not only through Mφ polarization but also via recruitment/migration and proliferation of Mφ and their precursor, monocytes. We therefore aim to clarify these previously unstudied aspects of the secondary paracrine effect in a systematic manner using hAM-MSCs.

RESULTS

hAM-MSC-dressing therapy was feasible, safe, and effective to treat ICM in a rat model

We characterized hAM-MSC-dressing therapy (epicardial placement of hAM-MSC-dressing) in a rat post-MI ICM model to progress our development of this emerging technique toward clinical application. This human-to-rat xenogeneic cell transplantation model without the use of an immunosuppressive reagent has been validated.[10] At 4 weeks after left coronary artery ligation, Lewis rats received epicardial placement of a 1-cm^2 hAM-MSC-dressing (hMSC-dressing group) or a same-sized fibrin sealant film with no cells (no cell-dressing group). The hAM-MSC-dressing was produced using a fibrin sealant film (TachoSil; Takeda) and 2×10^6 hAM-MSCs, as we previously optimized and reported.[9] An additional group received sham treatment (open and close of the chest only) at 4 weeks post-MI to serve as a control (ICM group). Production, handling, and epicardial placement of a hAM-MSC-dressing were straightforward and sufficiently completed in all rats studied. There was no obvious complication related to hAM-MSC-dressing therapy.

At day 28 after treatment, echocardiography demonstrated improved left ventricular ejection fraction (LVEF) post-hAM-MSC-dressing therapy compared to both no cell-dressing and ICM control groups (Figure 1A). LV end-systolic volume (LVESV) was reduced in the hAM-MSC-dressing group compared to other groups. Epicardial placement of no cell-dressing had no effect on these parameters. Of note, improved cardiac function and structure in the hAM-MSC-dressing group persistently lasted for 90 days post-treatment (Figure 1B).

Macroscopic observation exhibited that the hAM-MSC-dressing structure was preserved and remained adhering to the heart surface at day 3 after epicardial placement, whereas it was not clear at day 28 (Figure 1C). Immunohistofluorescent studies revealed that a considerable number of donor hAM-MSCs (labeled with CM-DiI prior to transplantation) were present on the epicardial surface of the infarct and peri-infarct areas at day 3 after transplantation, with the number reduced by day 28. The majority of transplanted hAM-MSCs remained on the heart surface without migration into the myocardial tissue. Furthermore, quantitative assessment of donor cell survivals using real-time PCR analysis for the primate specific *ALU* gene demonstrated that the average of 79.1% of the total transplanted hAM-MSCs remained at the LV walls of immunocompetent rats at day 3 post-transplantation. However, by day 28, only a very small percentage (<1%) of hAM-MSCs was retained in the heart (Figure 1D).

We then assessed the infiltration of $CD45^+$ cells into the hAM-MSC-dressing construct to investigate the local immunological response. The results demonstrated that the number of accumulated $CD45^+$ cells appeared to be larger in the hMSC-dressing group, compared to implantation of a film incorporating no cells, at both days 3 and 28 post-treatment, whereas the changes are not statistically significant (Figures S1A and S1B). In addition, the level of myocardial *Tnfa*, a major inflammatory cytokine, was not changed between the hMSC-dressing and sham ICM groups (Figure S1C).

These data collectively suggest that immunological response and rejection against hAM-MSC-dressings in the rat model were mild, allowing transplanted hAM-MSCs to survive and function in the early/acute phase, whereas such immune response persistently continued by day 28, eventually rejecting the most of donor cells. Of note, this temporal survival of hAM-MSCs was sufficient to improve cardiac function and structure in a rat ICM model, proposing the utility of

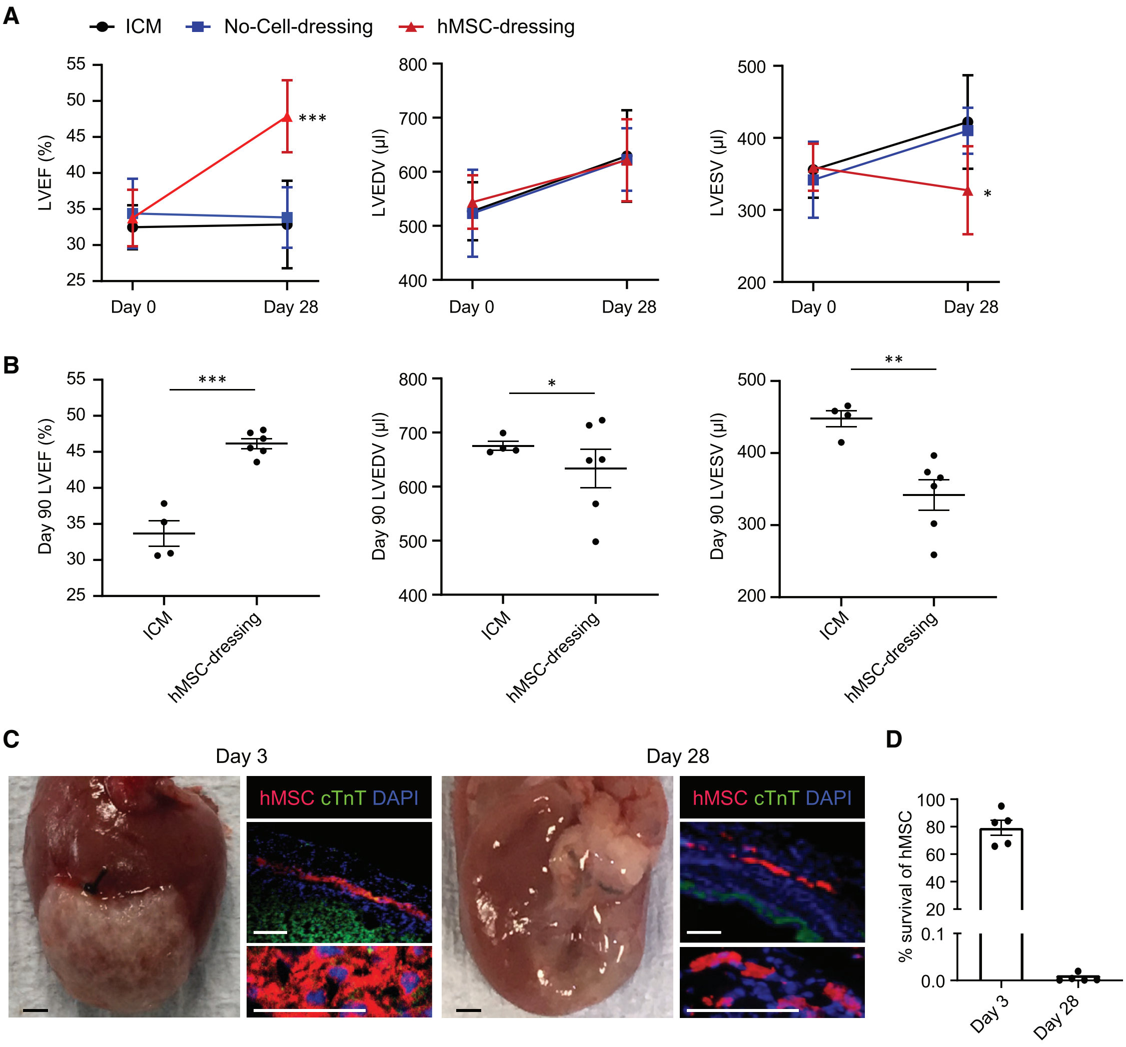

Figure 1. Improved cardiac function by hAM-MSC-dressing therapy in a rat ICM model

4 weeks after left coronary artery ligation in rats, a hAM-MSC-dressing (fibrin sealant film containing 2×10^6 hAM-MSCs; hMSC-dressing group), a fibrin sealant film only (no cell-dressing group), or nothing (no treatment; ICM group) were placed onto the heart surface. (A) Cardiac function and dimensions assessed by using echocardiography before treatment (day 0 = 4 weeks post-MI) and day 28 post-treatment. LVEF, left ventricular ejection fraction; LVEDV, left ventricular end-diastolic volume; LVDSV, left ventricular end-systolic volume; n = 7–10 in each group. *p < 0.05 and ***p < 0.001 versus both ICM and no cell-dressing groups. One-way ANOVA with Tukey's post hoc tests for multiple comparisons. Data presented as mean ± SEM. (B) Echocardiography indexes measured at day 90 post-treatment showing a longer-term effect of hAM-MSC-dressing therapy. *p < 0.05, **p < 0.01, and ***p < 0.001. n = 4–6 in each group. Student's t test. (C) Macroscopic and immunohistolabeling images of the hearts collected at days 3 and 28 post-hAM-MSC-dressing therapy. Immunolabeling samples were co-stained for cardiac troponin T (cTnT) and 4′,6-diamidino-2-phenylindole (DAPI). Donor hAM-MSCs were labeled with CM-Dil (red) before transplantation. Scale bars, 1 mm or 100 µm for macro- and micro-images. (D) Quantitative cell retention of hAM-MSCs in the rat LV tissue assessed by analyzing genomic DNA (gDNA) levels of the primate-specific *ALU* gene by qPCR at days 3 and 28 after epicardial placement of hAM-MSC-dressing (including 2×10^6 hAM-MSCs). n = 5 hearts with 3 technical replicates in each time point.

this therapeutic approach as a clinical treatment and also justifying this model to be used for the following mechanistic investigations of hAM-MSC-dressing therapy.

hAM-MSC-dressing therapy enhanced myocardial tissue repair

We could not find any CM-DiI-labeled donor MSC that was also positive for cardiac troponin T (cTnT) post-hAM-MSC-dressing therapy (Figure 1C), agreeing that the mechanism by which this treatment improved cardiac function was not cardiomyogenic trans-differentiation of donor MSCs. Thus, histological myocardial repair was further investigated post-hAM-MSC-dressing therapy. Picrosirius red staining showed that pathological interstitial fibrosis, which is a major component of adverse ventricular remodeling post-MI[25], was reduced in the peri-infarct and remote myocardial areas at day 28 after post-hAM-MSC-dressing therapy compared to the ICM control (Figure 2A). The reduced fibrosis was particularly evident in the

peri-infarct area. Wheat germ agglutinin staining clarified that hypertrophy of surviving cardiomyocytes, another important factor in post-MI ventricular remodeling, was attenuated in the peri-infarct area of the hAM-MSC-dressing-treated rats (Figure 2B). We also observed that microvascular formation in the peri-infarct myocardium was enhanced by the hAM-MSC-dressing therapy, as assessed by using isolectin B4 histological staining (Figure 2C).

These findings of histological myocardial repair post-hAM-MSC-dressing therapy were associated with upregulation of reparative genes, including *rIl10*, *rCxcl12*, *rIgf1*, *rTgfb1*, *rVcam1*, *rIl6*, and *rFgf2*, assessed by quantitative real-time PCR of the LV myocardium at day 3, compared with both the ICM group and normal heart group (Figure 2D). On the other hand, some known reparative or angiogenic factors, i.e., *rVegfa* and *rHgf* genes, were not upregulated significantly. Furthermore, we were also able to detect sizable expression of the similar profile of human genes involved in myocardial repair, including *hIL10*, *hCXCL12*, *hIGF1*, *hTGFB1*, and *hVCAM1*, in the same rat LV tissue with hAM-MSC-dressing therapy (Figure S2). Expression of these genes in the rat heart tissue were markedly upregulated when compared to the expression in *in vitro*-cultured hAM-MSCs.

Reparative gene upregulation by hAM-MSC-dressing therapy mostly came from host rat cells

Our next aim was to clarify the ratio of the source of reparative factor expression post-hAM-MSC-dressing therapy between the host rat cells versus donor human cells. Therefore, we examined the above-collected rats' LV tissues transplanted with hAM-MSC-dressings by using human- and rat-specific quantitative real-time PCR primers and absolute DNA copy number analysis.

To assess the ratio of human:rat DNA on day 3 after hAM-MSC-dressing therapy in the ICM rat heart, genomic DNA (gDNA) was extracted from the LV, and the samples were quantified using the primate-specific *ALU* TaqMan primer, which is expressed in human cells but not in rat cells. Cycle threshold (C_T) values were compared against a DNA standard curve containing a dilution series of known concentrations of human DNA mixed with rat DNA. The result demonstrated that human-derived DNA contributed to 2.3% of the total DNA extracted from the xenogeneic sample (rat LV myocardium with hAM-MSC-dressing) at day 3 post-transplantation (Figure 3A). Subsequently, human and rat gDNA templates were employed to construct a standard curve using known amounts of DNA copies ($1 \times 10^7 - 1 \times 10^2$), as previously described.[26–28] These standards were used to calculate human and rat gene transcript copies in 10 ng cDNA isolated from the rat LV tissue with hAM-MSC-dressing therapy (Figure 3B). The quantity of each transcript obtained by real-time PCR was normalized with the internal reference gene human and rat *GAPDH*, respectively. The mRNA copies calculated by qPCR analysis demonstrated that far-greater proportions of expression of these reparative genes came from the host rat cells rather than the donor hAM-MSC. Rat *Igf1* was found to be around 3 logs higher compared to human *IGF1*. Rat *Il10*, *Cxcl12*, *Tgfb1*, and *Vcam1* were all greater than 2 logs higher compared to their human equivalent. These data suggest that the vast majority of the paracrine mediators contributing to myocardial repair post-hAM-MSC-dressing therapy is from the host cardiac endogenous cells rather than the transplanted donor MSCs.

M2Mφ accumulation was markedly augmented by hAM-MSC-dressing therapy

We then aimed to identify the cell type(s) within the rat heart that upregulated the above-mentioned tissue-repair genes in response to hAM-MSC-dressing therapy. Immunohistolabeling showed a marked increase in cells positive for CD206, a common M2Mφ marker,[29] in both the infarct and peri-infarct regions at day 3 after hAM-MSC-dressing therapy compared to the ICM control (Figure 4A). The most evident increase in $CD206^+$ M2Mφ was observed in the infarct area post-hAM-MSC-dressing therapy. This enhanced accumulation of $CD206^+$ M2Mφ in the damaged myocardium was confirmed at day 10 (Figure 4B) and day 28 (Figure 4C) after treatment. Consistent to these histological findings, quantitative real-time PCR demonstrated that *rCd206* was upregulated in the heart at day 3 after hAM-MSC-dressing therapy compared to the ICM control and normal heart groups (Figure S3). On the other hand, the expression level of *rCd86*, a marker for M1-like Mφ phenotype,[22] was not significantly different between the hAM-MSC-dressing and ICM groups. Together, these data suggest that hAM-MSC-dressing therapy increases the abundance of $CD206^+$ M2Mφ, which are likely to serve as the mediator of the hAM-MSC-induced secondary paracrine effect for myocardial tissue repair.

hAM-MSCs augmented the M2Mφ abundance in a paracrine manner *in vitro*

To further investigate the ability of hAM-MSCs to increase the number of M2Mφ, we set up an *in vitro* co-culture experiment, in which hAM-MSCs were cultured in a Transwell with mouse bone marrow-derived Mφ (unstimulated; M0) cultured in the lower chamber. This non-contact co-culture model allowed for the investigation of the hAM-MSC's paracrine signal that would affect Mφ characters. After 48 h of the co-culture, flow cytometry analysis found that the ratio and absolute number of $CD11b^+F4/80^+CD206^+$ M2Mφ were both increased compared to the ordinary Mφ culture (Figures 5A and S4). An immunocytolabeling study confirmed these results: an increased number of Mφ were double positive for CD206 and F4/80 after co-culture with hAM-MSCs (Figure 5B). In addition, increased CD206 protein expression in Mφ by hAM-MSC co-culture was confirmed by using western blot analysis (Figure S5). The degree of this CD206 increase was similar to that by IL-4 treatment, which is known to strongly induce M2Mφ polarization.[30]

A reparative, M2-like phenotype of mouse-derived $CD206^+$ Mφ induced by non-contact co-culture with hAM-MSCs was confirmed by using quantitative real-time PCR analysis (Figure 5C). Expression of M2Mφ markers, *mCd206*, *mArg1*, and *mFizz1*, was markedly upregulated after co-culture with hAM-MSCs. *mTgfb1* was also

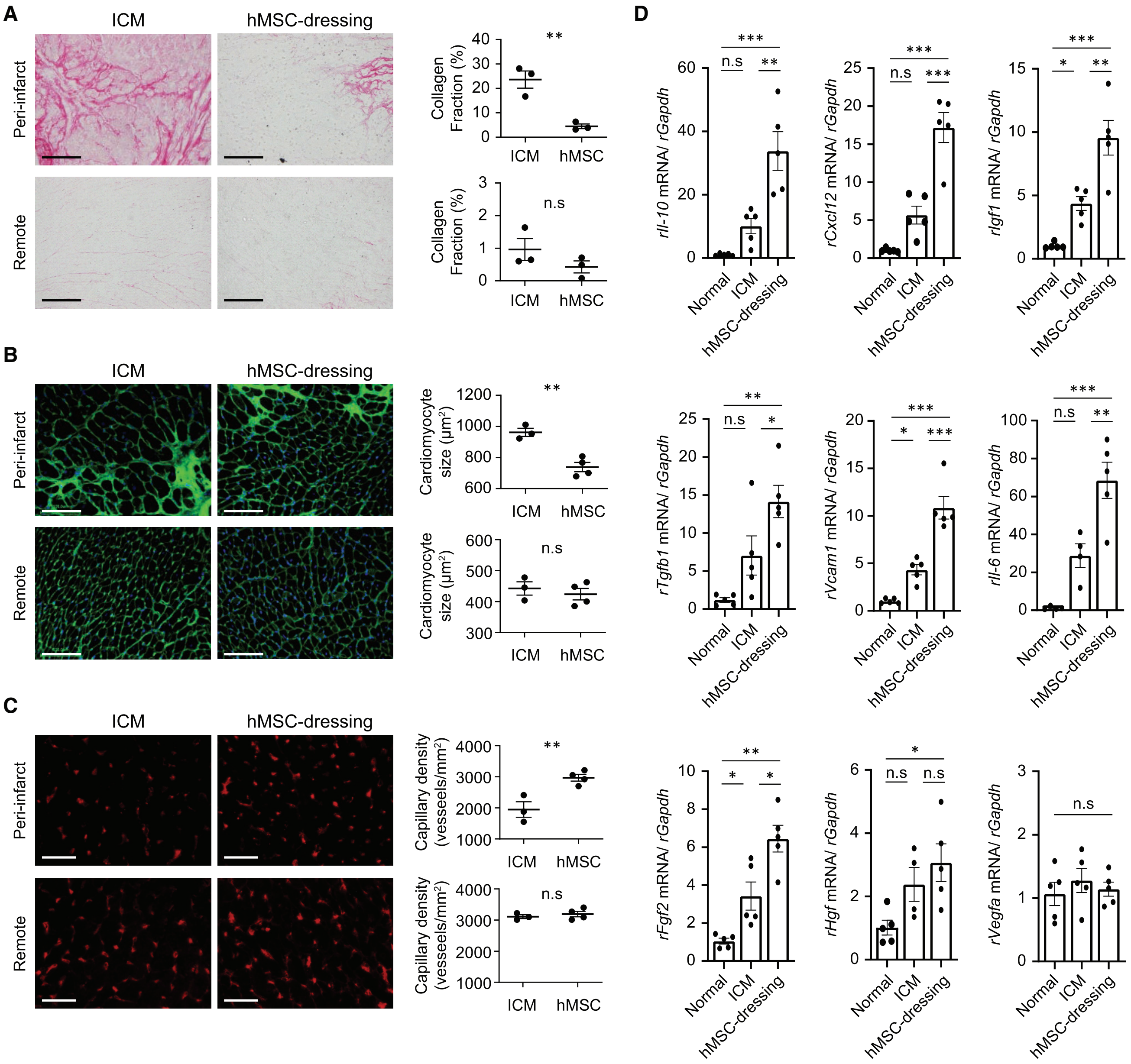

Figure 2. Augmented myocardial tissue repair by hAM-MSC-dressing therapy

Rat hearts were collected at day 3 or 28 after hAM-MSC-dressing therapy or no treatment (ICM group) in a rat ICM model and analyzed for cardiac tissue repair. (A) Picrosirius red staining images quantifying interstitial fibrosis at 28 days post-treatment. Collagen fraction was measured in the infarct and per-infarct border zone separately, and the data are presented in the charts. Scale bars, 100 μm. n = 4–5 rats in each group. (B) Wheat germ agglutinin (green) and (DAPI; blue) staining exhibiting the cardiomyocyte size at 28 days post-treatment. The cross-sectional area of at least 50 cardiomyocytes in each of the peri-infarct and remote areas per rat was measured, and the data are presented in the charts. Scale bars, 100 μm. n = 4–5 rats in each group. (C) Isolectin B4 staining showing the capillary vessels (red) at day 28 post-treatment. Capillary density was measured in the infarct and per-infarct border zone separately, and the data are presented in the charts. Scale bars, 100 μm. n = 4–5 rats in each group. (D) Quantitative real-time PCR analysis data showing increased expression of multiple rat reparative genes of the rat LV samples of the hAM-MSC group, as compared to the ICM group, at day 3 post-treatment. The normal (no MI, no treatment) group was included as a reference. n = 5 hearts in each group. Data are presented as mean ± SEM. *p < 0.05, **p < 0.01, and ***p < 0.001; n.s, not significant. Student's t test for statistical analysis (A–C); one-way ANOVA with Tukey's post hoc tests (D).

upregulated by the co-culture with hAM-MSCs. On the other hand, *mInos*, *mIl1b*, *mTnfa*, and *mCd86* were downregulated by co-culture. There was an upregulating tendency in expression of tissue-repair-related genes, including *mIgf1* and *mCxcl12*, but these changes were not significant statistically. Increases in interleukin (IL)-10 and transforming growth factor β1 (TGF-β1) protein secretion were observed

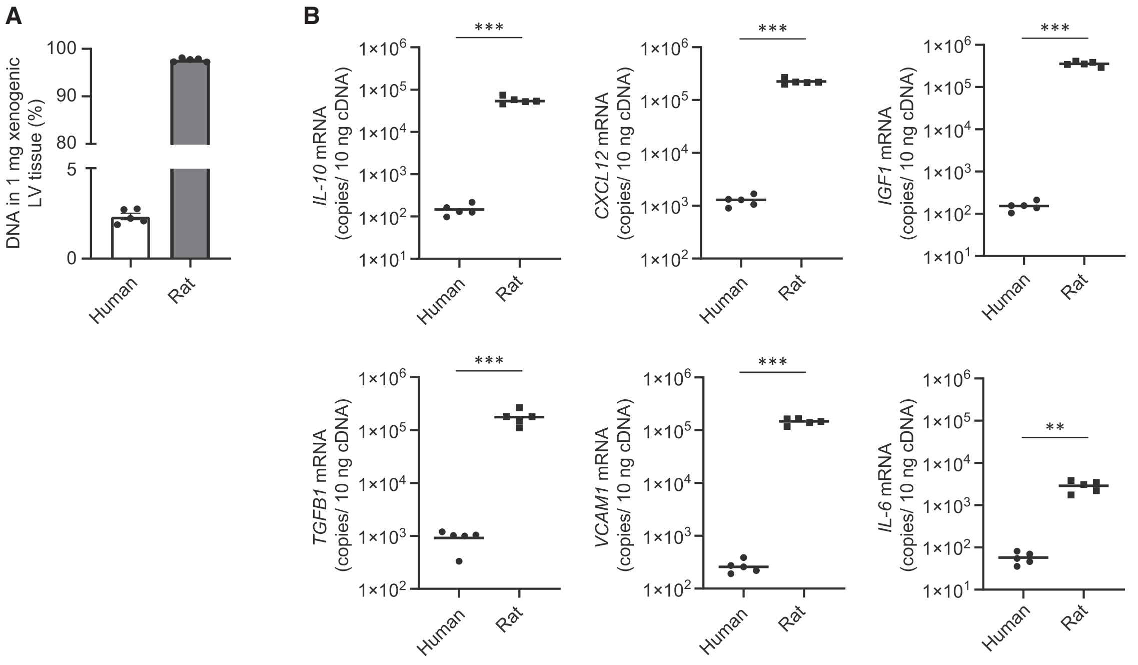

Figure 3. Donor- and host-derived reparative genes in the rat heart after hAM-MSC-dressing therapy

The rat ICM hearts were explanted at 3 days after transplantation of 2×10^6 hAM-MSCs by the-dressing method. gDNA was extracted from 25 mg of LV tissue, and 100 ng DNA was amplified by qPCR. (A) Ratio of human and rat DNA contained in the xenogeneic sample. Mean *ALU* quantification cycle (Cq) values were used to calculate the ratio of human gDNA in each heart gDNA sample according to a 10-fold dilution series of known ratios of rat heart gDNA and hAM-MSC gDNA. n = 5 with 3 technical replicates. (B) The number of gene transcript copies in 10 ng cDNA isolated from the hAM-MSC-dressing group. Expression levels of human and rat reparative genes were determined in ischemic LV tissue of the hAM-MSC-dressing group utilizing gDNA standard curves of 1×10^7–1×10^2 gene copies and species-specific primer pairs (human versus rat) in the xenogeneic sample. Data points presented as mean (log10). n = 5 with 3 technical replicates. $^{**}p < 0.01$ and $^{***}p < 0.001$. Student's t test.

in the supernatant of Mφ + hAM-MSC co-culture compared to mono-culture of Mφ or hAM-MSC (Figure 5D). In addition, a flow cytometry study using fluorescence-labeled immunoglobulin G (IgG)-coated beads demonstrated that the phagocytotic activity of Mφ was increased by co-culture with hAM-MSCs (Figure S6). The degree of the increased phagocytosis activity was equivalent with that of Mφ stimulated with IL-4 and lipopolysaccharide (LPS), which are both reported to enhance Mφ phagocytosis.[31] All of these results indicate that secretion from hAM-MSCs promotes the production of M2Mφ from Mφ, which exhibit enhanced potential of anti-inflammation and myocardial repair.

hAM-MSCs drove Mφ polarization to an M2 phenotype through prostaglandin E_2 (PGE_2)

There are multiple likely mechanisms to increase the accumulation of M2Mφ in the heart *in vivo*, including driven M2 polarization, augmented proliferation, and increased recruitment of Mφ or monocytes (Mφ precursor). hAM-MSC's paracrine effects on these mechanisms are not fully identified. First, we aimed to detect the major mediator(s) derived from hAM-MSCs to accelerate the mouse Mφ polarization in the *in vitro* co-culture model using neutralizing antibodies to human IL-10, TGF-β1, insulin growth factor 1 (IGF1), CXCL12, and CCL2. These factors have been reported to promote M2 polarization.[20,23,32,33] Real-time PCR found that upregulation of *mCd206* and *mArg1* in Mφ by hAM-MSC co-culture was eliminated by blocking CXCL12 and CCL2 (Figure S7A). Downregulation of *mTnfa* by hAM-MSC coculture was also abolished by these blockings. However, flow cytometry analysis of these Mφ populations did not show significant reduction of $CD11b^+F4/80^+CD206^+$ cells by inhibition of CXCL12 or CCL2 compared to control IgG antibodies (Figure S7B). Inhibition of IL-10, TGF-β1, or IGF1 showed no effect on hAM-MSC-induced Mφ polarization as measured by either PCR or flow cytometry analysis.

Subsequently, we investigated the role of PGE_2, which is another likely factor to moderate Mφ polarization and was robustly secreted by hAM-MSCs (Figure 6A). PGE_2 is generated from arachidonate through a COX-2-dependent pathway. To inhibit the PGE_2 signaling, a selective COX-2 inhibitor NS-398[34] was supplemented in the co-culture model. Flow cytometry analysis revealed that NS-398 treatment reduced the hAM-MSC-induced increase in the ratio of $CD11b^+F4/80^+CD206^+$ cell occurrence (Figure 6B). Real-time PCR analysis demonstrated that blockage of PGE_2 via COX-2 inhibition attenuated the hAM-MSC-induced upregulation of *mCd206*, *mArg1*, *mFizz1*, and *mTgfb1* compared to the control (Figure 6C). Together, these findings suggest that PGE_2 is a major mediator in hAM-MSC-induced polarization of Mφ to an M2 phenotype.

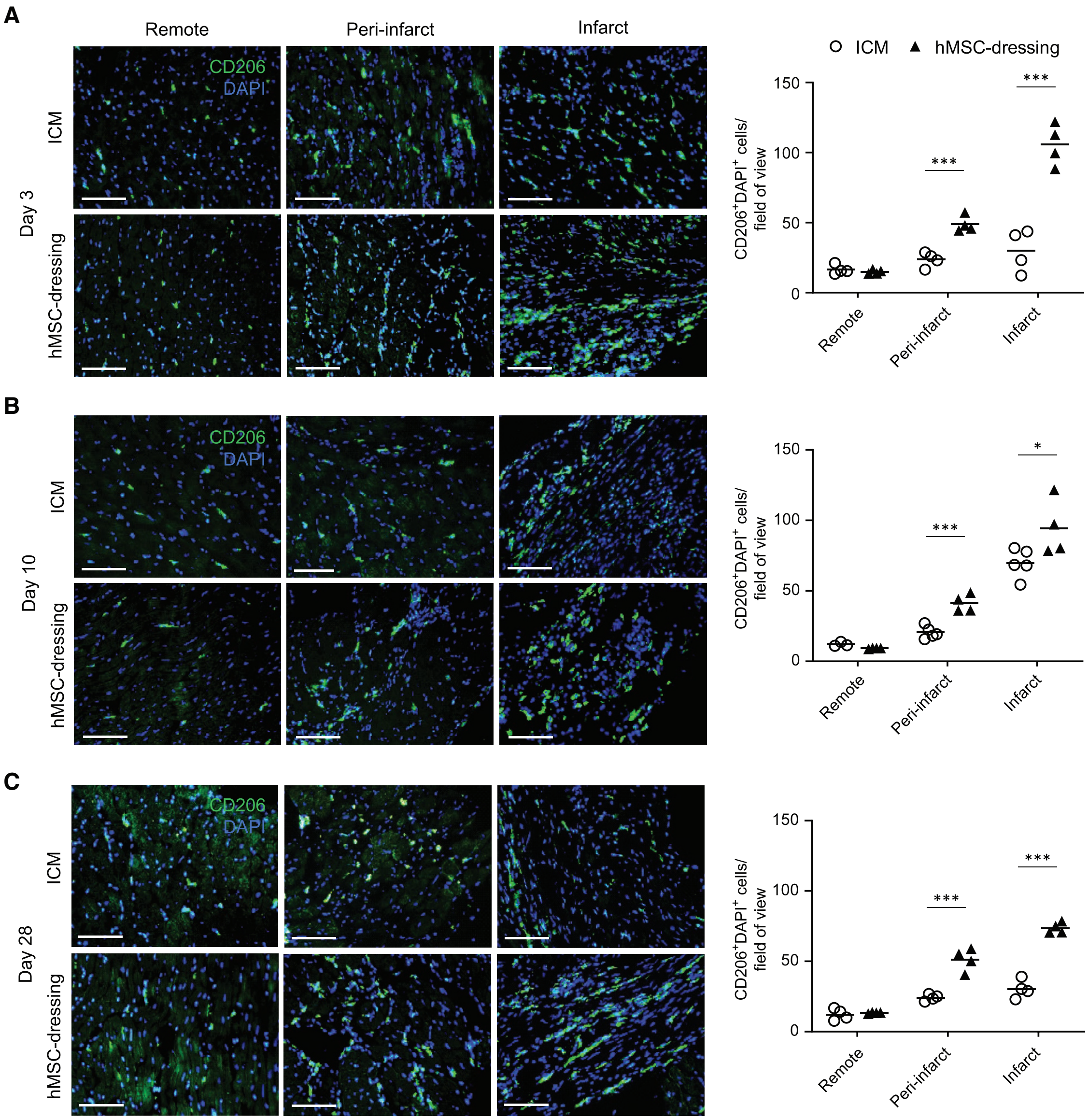

Figure 4. Enhanced M2Mφ numbers by hAM-MSC-dressing therapy *in vivo*

(A–C) Representative immunohistolabeling images demonstrating that accumulation of $CD206^+$ cells (green) in the remote, peri-infarct, and infarct regions of the myocardium at (A) 3 days, (B) 10 days, and (C) 28 days post-hAM-MSC-dressing therapy (hMSC-dressing group), in comparison with the ICM control group. Samples were counter stained with DAPI. The number of $CD206^+DAPI^+$ cells was counted and presented in the charts. At least 10 images per myocardial area were assessed per animal. Scale bars, 50 μm. Data are presented as mean value per heart. n = 4–6 hearts in each group. *$p < 0.05$ and ***$p < 0.001$ versus ICM group. Student's t test.

hAM-MSCs increased proliferation of Mφ through CCL2 and TGF-β1

Next, the role and mechanism of hAM-MSCs to regulate local proliferation of Mφ were investigated using the same *in vitro* indirect co-culture model with antibody neutralization. It was observed that co-culture with hAM-MSCs increased the yield of mouse bone marrow-derived Mφ compared to mono-culture (Figure 7A). Immunofluorescent staining found that co-culture with hAM-MSCs elevated the number of Mφ expressing a proliferation marker Ki67 (Figure 7B). Antibody neutralization of human CCL2 and TGF-β1 eliminated the increased proliferation by co-culture with hAM-MSCs, compared to IgG control antibody (Figure 7C), suggesting a role for these factors in the hAM-MSC-enhanced proliferation of Mφ. Blocking human IL-10, CXCL12, or IGF1 with neutralizing antibodies or inhibition of PGE_2 using NS-398 had no effect on hAM-MSC-induced proliferation of Mφ (Figure S8). These findings collectively suggest that CCL2 and TGF-β1 secretion from hAM-MSCs contributes to the increased proliferation of Mφ.

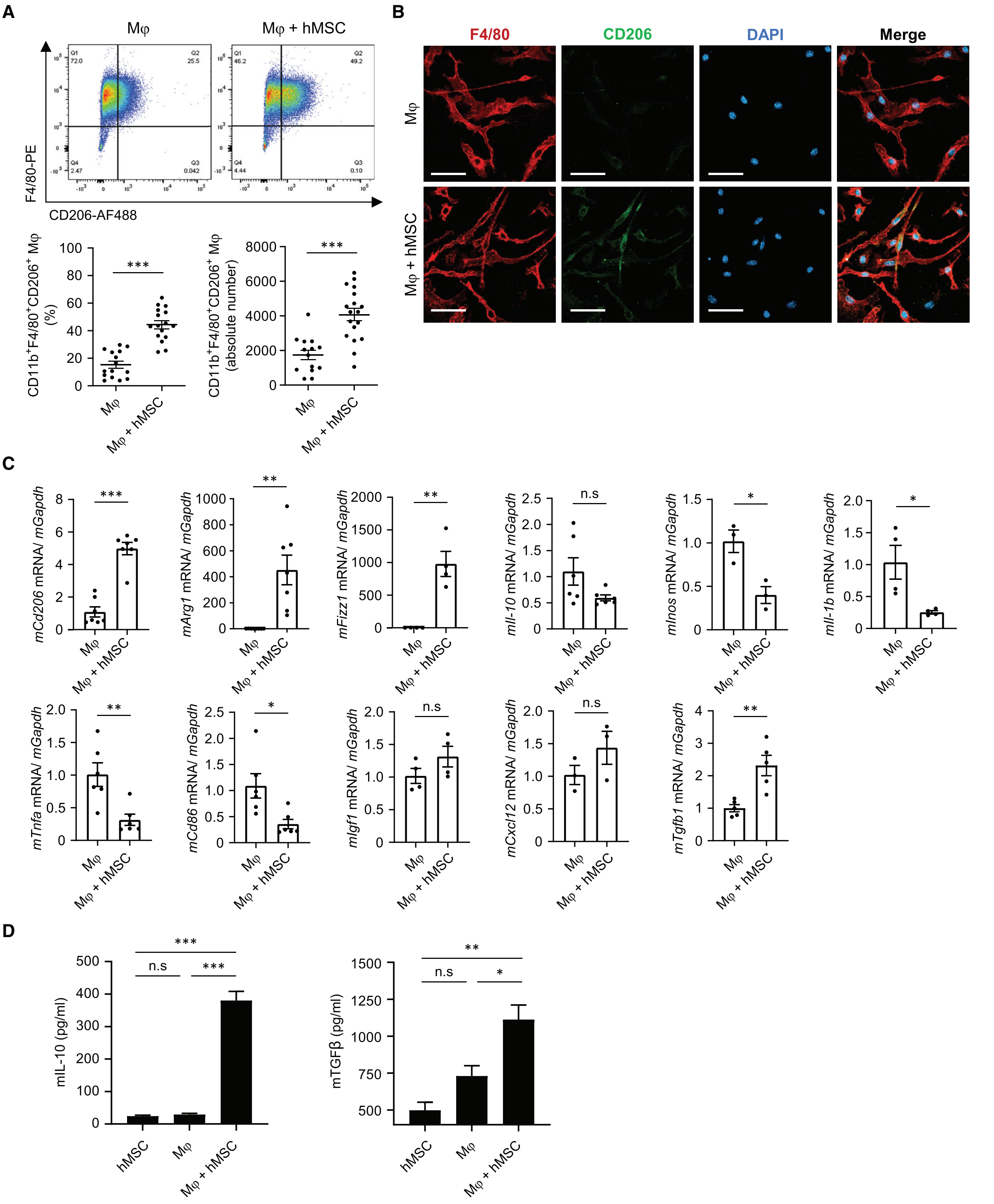

(legend on next page)

hAM-MSCs enhanced migration of Mφ and monocytes via CCL2 signaling

Recruitment or migration of Mφ or monocytes to the site of injury will influence accumulation of M2Mφ at the damaged myocardium; therefore, the ability of hAM-MSC to promote migration of these cells was explored as a mechanism of increasing M2Mφ numbers. Freshly isolated mouse bone marrow-derived monocytes or Mφ (M0) produced from these monocytes were seeded in a Transwell chamber and allowed to migrate through the membrane toward a confluent hAM-MSC culture or media-only control in the lower chamber over a 2-h period. The number of migrated cells through the Transwell membrane was counted. As a result, we observed that a small number of monocytes and Mφ migrated through the membrane in the media-only control group, and this migration was increased approximately 4- to 6-fold by co-culturing with hAM-MSCs (Figure 8). As CCL2 is a potent activator of monocyte migration,[35] we tested the function of CCL2 on the hAM-MSC-induced migration of monocytes and Mφ. Indeed, antibody neutralization of human CCL2 attenuated the hAM-MSC-induced increase of migrated monocytes and Mφ, whereas administration of CCL2 recombinant protein (instead of hAM-MSC co-culture) increased their migration. These data indicate that CCL2 secreted by hAM-MSCs regulates migration/recruitment of monocytes and Mφ.

DISCUSSION

As an important step to develop AM-MSC-dressing therapy toward clinical application, this study characterized the feasibility, safety, and efficacy of hAM-MSC-dressings in the rat ICM model. Fabrication and epicardial placement of hAM-MSC-dressings were straightforward and safe. After epicardial placement, hAM-MSCs survived adequately in an immunocompetent rat and robustly enhanced cardiac performance in correspondence to augmented myocardial tissue repair. An increase in LVEF by the hAM-MSC-dressing was 15.0% (from 34.0% ± 0.9% pre-treatment to 49.0% ± 1.2%), which is more than equivalent compared to that by a syngeneic rat AM-MSC-dressing in the same rat ICM model (7.4% increase from 36.8% ± 1.7% to 44.2% ± 2.6%),[9] ensuring a substantial therapeutic efficacy of hAM-MSC-dressings. Also, survival of hAM-MSCs in the xenogeneic rat heart (79.1% at day 3) was compatible to that of syngeneic rat AM-MSCs survival of 62.5%,[9] assuring the usage of hAM-MSCs in allogeneic treatment in the clinical arena. As the hAM-MSC-dressing technique requires exposure of the heart through thoracotomy, it will be sensible to perform this treatment in the late-phase post-MI, i.e., ICM, particularly in conjunction with coronary artery bypass graft (CABG). The enhanced outcome of CABG by adding cell-based therapy has been reported,[36] and an addition of MSC-dressing therapy to CABG is technically straightforward. The MSC-dressings can be instantly produced within the surgical theater without requirement for a cell-processing facility in the hospital. Also, it is possible to perform MSC-dressing therapy solely for ICM patients through a small incision (without CABG). Thus, MSC-dressing therapy has a great potential to become a widely adopted treatment of ICM.

The current concept of the mechanism underpinning the MSC-induced therapeutic effect is the direct paracrine effect, which is induced by the secretome from donor MSCs that stimulate host cardiac cells, directly resulting in neovascular formation, anti-fibrosis, and cardiomyocyte protection in the infarcted and peri-infarct myocardium.[12] However, following epicardial placement of MSCs, the majority of donor MSCs retains on the heart surface, and thereby, whether their reparative secretion adequately reaches to the deep inside of myocardium, particularly in large animals or human patients, has been questionable. The human-to-rat xenogeneic cell transplantation model used in this study advocated an answer to this uncertainty. Quantitative PCR analysis of species-specific gene copy numbers in the rat heart post-hAM-MSC-dressing therapy clearly revealed that the origin of the up-regulated reparative factors, including IL-10, CXCL12, IGF1, and TGFβ-1, was rat endogenous cardiac cells, with a negligible proportion of signal from the hAM-MSCs. This strongly supports the theory of the secondary (indirect) paracrine effect, which means that donor MSCs secrete the factors that stimulate host cells to release reparative cytokines, growth factors, and/or exosomes, inducing myocardial tissue repair and eventually leading to improved cardiac function post-MI.[12,14,37] In addition, the multiple pieces of information from this and other studies propose that cardiac M2Mφ is the key mediator of the secondary paracrine effect. Such data include the following: (1) the occurrence of cardiac M2Mφ robustly increases post-hAM-MSC-dressing therapy in the wide area of the heart; (2) the profile of upregulated reparative genes post-hAM-MSC-dressing therapy, i.e., *IL-10*, *CXCL12*, *IGF1*, and *TGFB1*, closely corresponds to that in cardiac M2Mφ reported;[38] and (3) M2Mφ play a critical role in post-MI cardiac repair, and forced augmentation of M2Mφ by IL-4-based treatment or adopted transfer results in enhanced myocardial repair post-MI in mice.[32,38,39] Of note, this concept is able to explain why hAM-MSCs localized on the heart surface after the dressing method can repair the myocardium far from their localization. Epicardially localized hAM-MSCs increase the number of M2Mφ through their secretion initially in the vicinal areas. Then, these M2Mφ dynamically

Figure 5. Increased M2Mφ by co-culture with hAM-MSCs *in vitro*

Mouse bone marrow-derived Mφ were co-cultured with hAM-MSCs using a Transwell insert (0.4 μm pore size) for 48 h to investigate the role of hAM-MSC secretome on Mφ activation. (A) Representative flow cytometry dot plots of Mφ with or without co-culture with hAM-MSCs (Mφ + hMSC and Mφ groups, respectively). Cells double positive for F4/80 and CD206 within the $CD11b^+$ population were defined as M2Mφ. Percentage of M2Mφ among the total live cells as well as the absolute number of M2Mφ was calculated and presented in the charts. (B) Immunofluorescent images for F4/80, CD206, and DAPI of Mφ, demonstrating enhanced CD206 expression by co-culture with hAM-MSCs compared to the ordinary Mφ monoculture. Scale bars, 20 μm. (C) RT-PCR quantification of Mφ gene expression after hAM-MSC co-culture (Mφ + hMSC group) compared to the Mφ-only group using *GAPDH* as an internal reference gene. Fold change in mRNA levels was normalized to 1 for the control group. n = 4 biological replicates with 3 technical replicates. (D) Secretion levels of IL-10 and TGF-β1 of Mφ with or without co-culture with hAM-MSCs. n = 4 biological replicates with 3 technical replicates. Data are shown as mean ± SEM. *p < 0.05, **p < 0.01, and ***p < 0.001. Student's t test (A and C); one-way ANOVA with Tukey's post hoc tests (D).

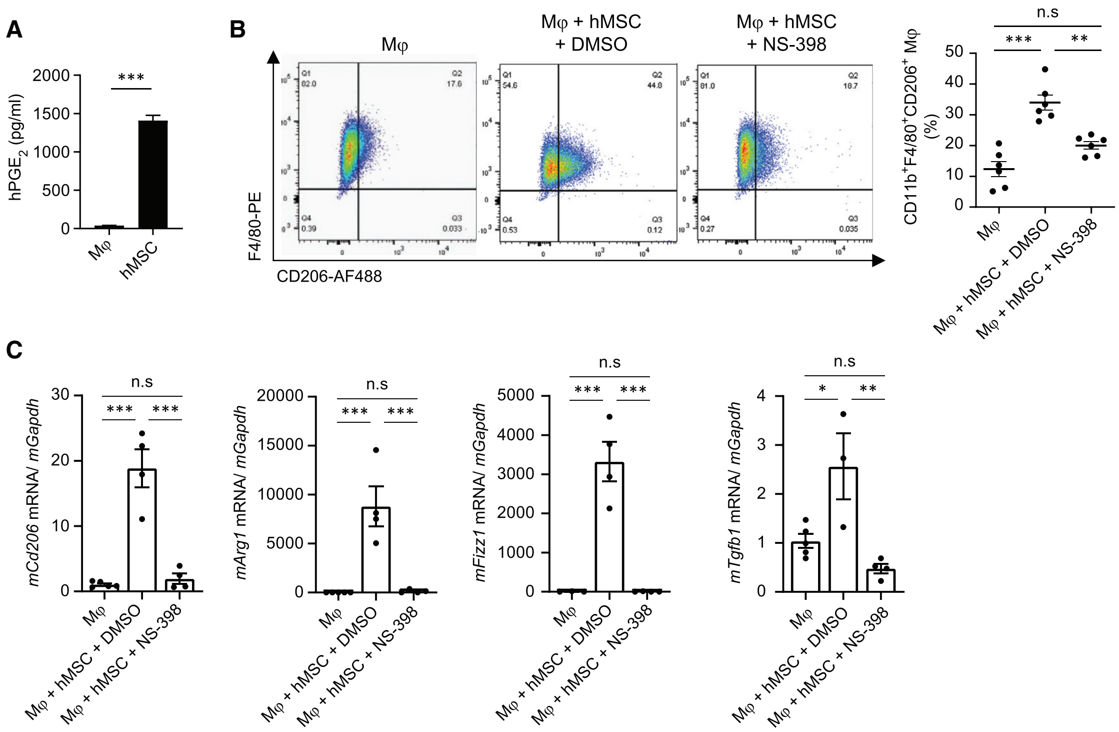

Figure 6. Augmented M2Mφ polarization by hAM-MSCs via a PGE_2 pathway

(A) PGE_2 expression in the supernatant of hAM-MSC culture (hMSC group) measured using ELISA. The DMEM group is normal (no cell) culture medium. n = 4. (B) Mouse bone marrow-derived Mφ were mono-cultured (Mφ group) or co-cultured with hAM-MSCs pre-treated with DMSO (vehicle control; Mφ + hMSC + DMSO group) or COX-2 inhibitor, NS-398, in DMSO (Mφ + hMSC + NS-398 group). After 48 h, Mφ were collected and assessed by flow cytometry, and the percentage of M2Mφ ($CD11b^+/F4/80^+/CD206^+$) from the total live cell population was quantified. n = 4–5 in each group. (C) Gene expression of Mφ in each group was measured by RT-PCR and normalized to the Mφ-only group. *Gapdh* was used as a reference gene. n = 3–5 biological replicates with 3 technical replicates. Data are presented as mean ± SEM. *$p < 0.05$, **$p < 0.01$, and ***$p < 0.001$. Student's t test (A) and one-way ANOVA with Tukey's post hoc test (B and C).

migrate throughout the heart, releasing reparative factors for tissue repair in the broader myocardial areas. The number of accumulated M2Mφ is much greater than that of surviving donor MSCs, and M2Mφ have a long (over months) lifetime in the heart. Therefore, M2Mφ offer a greater, broader, and more persistent reparative secretome, leading to more substantial and sustainable tissue repair in the extensive area of the heart. We are aware that this assumption should be confirmed using a model of specific depletion of M2Mφ in the heart *in vivo*; however, unfortunately, we have a technical limitation to do so with current research technologies. It has been reported that Mφ depletion using clodronate liposome administration reduces M2Mφ occurrence and attenuates MSC-induced paracrine effects.[18] However, we need to be careful in interpretation of the results of clodronate liposome-based studies, as clodronate liposome is not specific to M2Mφ, depleting all phagocytic Mφ phenotypes including M1-like pro-inflammatory Mφ. Development of a novel technology that exclusively depletes an increased M2Mφ subpopulation post-MSC therapy without causing inflammation or myocardial damage is warrantied.

From the results of this study and previous reports,[16,18,19] hAM-MSC transplantation, no doubt, increases the number of cardiac M2Mφ *in vivo*. We reproduced this finding in an *in vitro* indirect co-culture model using hAM-MSCs and mouse bone marrow-derived Mφ. hAM-MSC secretome increased $CD11b^+F4/80^+CD206^+$ Mφ, which also express other M2-specific markers, including *Arg1* and *Fizz1*. Their expression of inflammatory genes, *Inos*, *Il1b*, *Tnfa*, and *Cd86*, was downregulated, and their secretion of anti-inflammatory cytokines IL-10 and TGF-β1 was increased. Furthermore, this report suggests involvement of multiple different mechanisms by which hAM-MSC transplantation increases M2Mφ. First, consistent to previous findings, hAM-MSCs strongly drove Mφ polarization to a M2 phenotype, and this M2 polarization was dependent on PGE_2. The importance of PGE_2 secretion from MSCs in M2Mφ modulation in inflammatory disease settings, such as lung sepsis, arthritis, and diabetic CM, has been reported.[14,19] Second, proliferation of Mφ was increased by the hAM-MSC's paracrine signaling, and CCL2 and TGF-β1 played a role here. Third, hAM-MSC secretion increased migration of monocytes and Mφ, and CCL2 was responsible to this effect. Papa et al.[40] have reported in a mouse spinal cord injury model that MSC treatment increases Mφ recruitment and skews Mφ toward a neuroprotective M2-like phenotype via a CCL2 mechanism. Similarly, Takeda et al.[41] described MSC recruitment of anti-inflammatory monocytes and/or Mφ by CCL2 in a murine asthma model. Having said this, we cannot exclude involvement of other molecules by which hAM-MSCs increase

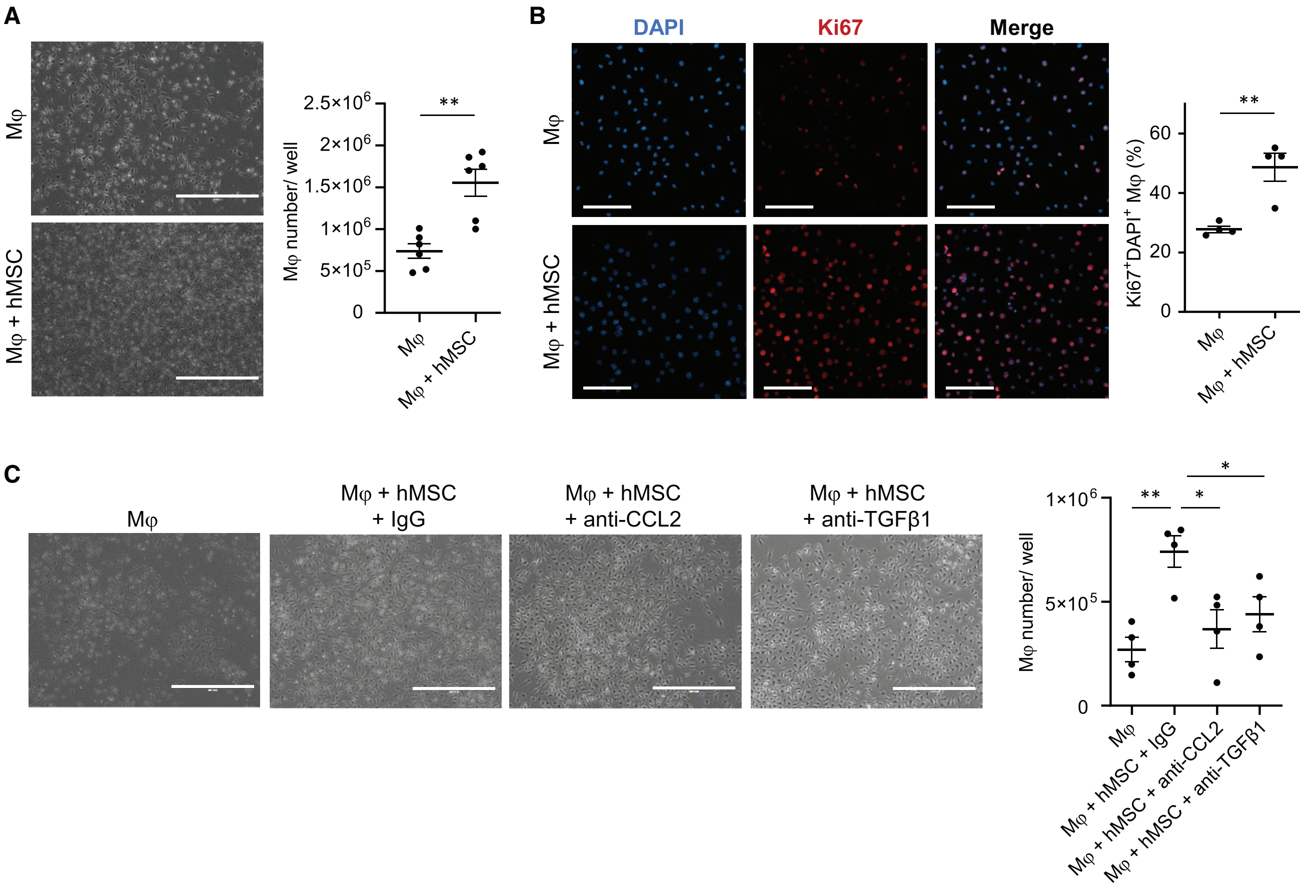

Figure 7. Enhanced Mφ proliferation by hAM-MSCs via CCL2 and TGF-β1 pathways

Mouse bone marrow-derived Mφ were cultured with or without hAM-MSCs in a non-contact co-culture model for 48 h. (A) Phase-contrast images showing the Mφ frequency. Mφ were dissociated for cell number counts, the results of which are presented in the chart. Scale bars, 400 μm. n = 6. (B) Immunocytolabeling showing Ki67 expression in Mφ. Collected Mφ were stained for a proliferation marker Ki67 and DAPI, and percentage of $Ki67^+$ nuclei was measured and present in the chart. Scale bars, 50 μm. n = 4 in each group. (C) Effects of inhibition of human CCL2 and TGF-β1 on hAM-MSC-mediated Mφ proliferation. Increased Mφ numbers by hAM-MSC co-culture were eliminated by addition of neutralizing antibodies for hCCL2 (Mφ + hMSC + anti-CCL2 group) and TGF-β1 (Mφ + hMSC + anti-TGF-β1 group). Isotype (IgG) antibody used as control. Representative phase-contrast images and a chart summarizing the data are presented. Scale bars, 400 μm. n = 4. Data are presented as mean ± SEM. $*p < 0.05$ and $**p < 0.01$. Student's t test (A and B) or one-way ANOVA with Tukey's post hoc test (C).

the M2Mφ abundance, including exosomes. Further comprehensive studies are needed to fully elucidate this complex biology, in which multiple cytokines, chemokines, microRNAs (miRNAs), and other factors are functioning in a complicated, coordinated manner.

This study focused on the examination of hAM-MSCs because these cells have important advantages as the donor for cell therapy in being easy to mass produce, in addition to the robust ability of enhancing myocardial repair.[2,10,11,42] hAM-MSCs are collected from clinical waste at the time of birth delivery; thus, no invasive biopsy is required to gather the cells. A larger number of peri-natal MSCs can be isolated from one amniotic membrane with a greater proliferative ability and less cellular senescence compared to adult tissue-derived MSCs. It is clear that the secondary paracrine effect described in this study is not specific to hAM-MSCs and is shared by other MSC types and non-MSC cells, as reported previously in a variety of models of cardiac and non-cardiac disease.[14–24,43,44] Having said this, there may be some cell-type specificities in this mechanism, although the major pathways are likely to be common. Further systematic comparisons of hAM-MSCs with other MSC types and non-MSC types may be interesting and will be one of the subjects of future investigations. In addition, this study did not include a dose-response study, as our previous study[9] has demonstrated a clear, positive correlation between the number of rat AM-MSCs and improvements in cardiac function within a cell dose ranging from 0 to 4×10^6 in the same model of MSC-dressing therapy in the rat ICM model. Given the comparable pattern and degree of myocardial repair and cardiac function improvement induced by hAM-MSCs (this study) and rat AM-MSCs,[9] it is likely that hAM-MSCs show a similar dose-response correlation as rat AM-MSCs. As the dose-response data from a rat study have a limitation in clinical translation, an appropriate dose-optimization study is warrantied in the future clinical trial.

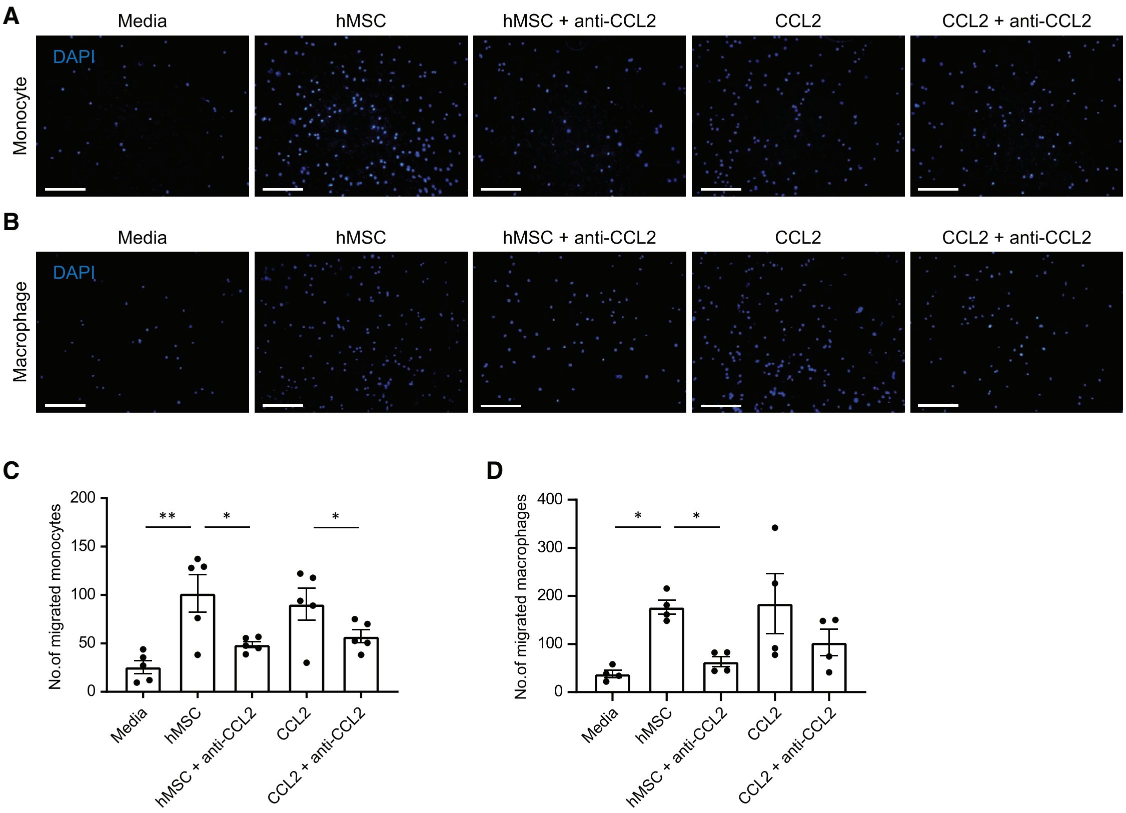

Figure 8. Enhanced migration of monocytes and Mφ by hAM-MSCs through CCL2

(A–D) Migration ability was assessed in freshly isolated mouse bone marrow-derived monocytes (A and C) or Mφ (B and D) by seeding in the upper well of a Transwell insert and measuring movement of these cells toward a confluent monolayer of hAM-MSCs (hMSC group) or no cell (media group) in the lower side of the membrane. Effects of co-culture with hAM-MSCs with human CCL2 neutralizing antibody (hMSC + anti-CCL2 group) and administration of recombinant CCL2 with or without anti-CCL2 antibody (CCL2 + anti-CCL2 and CCL2 groups) were also assessed. Migrated cells through the insert membrane were fixed and stained with DAPI and counted. Representative DAPI-staining images (A and B) and respective counts of migrated cell numbers are presented in the charts (C and D). Scale bars, 50 μm. n = 4–5 independent experiments. Conditions were repeated in duplicate for each biological replicate, and an average >5 frames per insert were captured. Data are presented as mean ± SEM. *p < 0.05 and **p < 0.01. One-way ANOVA with Tukey's post hoc test for multiple paired comparisons.

MATERIALS AND METHODS

Culture of hAM-MSCs

hAM-MSCs were kindly gifted by Dr. Yamahara and isolated from human fetal membranes, obtained during caesarean deliveries, as previously described.[9,42,45] Cells were cultured in alpha-minimal essential medium (αMEM; Gibco) containing 10% fetal bovine serum (FBS; Sigma-Aldrich), L-glutamine (200 mM; Gibco), and penicillin-streptomycin (100 U/mL; Sigma-Aldrich) at 37°C in a humidified atmosphere of 95% air and 5% CO_2.

hAM-MSC-dressing therapy in a rat ICM model

All assessments were carried out in a blinded manner wherever possible. MI was induced in female Lewis rats (175–200 g) by left coronary artery ligation under isoflurane anesthesia and mechanical ventilation as described previously.[9,46] The chest and skin were closed; then animals recovered. 4 weeks post-MI surgery, rats were subjected to echocardiography, and those presenting improper LVEF (>45% or <25%) were omitted from the experiment.[9,46] Included animals were randomly allocated into 3 treatment groups as described below. Hearts were re-exposed through a left thoracotomy, and the epicardial heart surface was exposed. As previously described, a fibrin sealant film of 1 cm^2 (TachoSil) loaded with 2×10^6 hAM-MSCs was placed on the heart surface covering ischemic areas[9] (hAM-MSC-dressing group; simplified to hMSC-dressing in figures). The ICM group received an open/close chest procedure only at 4 weeks after coronary artery ligation, whereas the no cell-dressing group was treated with TachoSil only (no donor cells). After each treatment, the chest was closed and the skin sutured.

Echocardiography

Transthoracic echocardiography was assessed pre-hAM-MSC transplantation following MI (baseline) and at day 28 and day 90 post-hAM-MSC transplantation by the Vevo-770 echocardiography machine (VisualSonics, Fujifilm, Netherlands) under isoflurane

anesthesia via a nose cone.[9,46–48] LV end-diastolic volume (LVEDV) and LVESV, under stable heart rate, were calculated using the area-length method.[49] LVEF was calculated as the following:

$$\text{LVEF}\ (\%) = (\text{LVEDV} - \text{LVESV})/\text{LVEDV} \times 100$$

All data were collected blind; n = 7–10 animals at day 28, and n = 4–6 animals at day 90 were measured with 3–5 technical replicates.

Histological analysis

Hearts were collected (n = 4–5 per group), fixed with 4% paraformaldehyde (PFA), and frozen in optimal cutting temperature (OCT) compound using liquid nitrogen. Cryosections were cut at a thickness of 6 μm using a Bright cryostat and then incubated with the following antibodies: polyclonal anti-cTnT (1:200; HyTest), monoclonal alpha sarcomeric actinin EA-53 (1:100; Thermo Fisher Scientific), polyclonal anti-CD206 (1:100; Santa Cruz Biotechnology), or monoclonal anti-CD45 (1:100; Abcam) at 4°C overnight. Conjugated WGA-AF488 (1:200; Invitrogen) was added for 1 h at room temperature. For isolectin B4 staining, biotinylated Griffonia simplicifolia lectin I-isolectin B4 (1:100 dilution vector L-1104) was used. These were followed by Alexa Flour-conjugated secondary antibodies (Thermo Fisher Scientific) for 1 h at room temperature and then counterstained using 1 ng/μ 4′,6-diamidino-2-phenylindole (DAPI). Images were captured by fluorescence microscopy (BZ8000; Keyence; 10× 20×, and 40× objectives) and analyzed using ImageJ software. For semiquantitative assessments, 5–10 fields per heart area (either infarct, peri-infarct, or remote [uninjured] areas) were randomly selected and assessed. To evaluate cardiomyocyte size, the cross-sectional area of cardiomyocytes (transversely cut; having central nuclei and surrounded by circle-shaped capillaries) was measured for 40 cardiomyocytes per area.[9,32,46]

In addition, PFA-fixed heart slices were incubated in 1.5% phosphomolybic acid (Sigma-Aldrich) for 30 min, 0.1% Sirius Red solution for 45 min, and then 0.5% of acetic acid (Sigma-Aldrich) solution for 3 min. Sections were washed three times for 5 min each. Sections were dehydrated through increasing concentrations of ethanol (70% and 100%) and xylene (20 s each immersion). Finally, sections were mounted using DPX mounting medium (VWR International). The wall thickness was measured at five independent regions of the infarct area. The quantity of the collagen fraction was calculated from five fields (BZ8000; Keyence; 20× magnification) of each area per heart using ImageJ software.

Collection of heart samples for qPCR and quantitative real-time PCR

At the chosen time after hAM-MSC transplantation, the rat LV tissue samples were collected by removing great vessels, atrium, and right ventricular (RV)-free wall from the collected hearts; n = 4–5 per group. The samples were snap frozen in liquid nitrogen and powdered for DNA and RNA extraction.

Quantitative assessment of donor cell presence

Donor cell survival was quantified by qPCR for the primate-specific *ALU* gene at days 3 and 28 post-hAM-MSC-dressing therapy (n = 5). gDNA was isolated from the rat LV samples using the AllPrep DNA/RNA Kit (QIAGEN). ALU TaqMan probe set (forward [fw]: 5′-GTCAGGAGATCGAGACCATCCT-3′; reverse [rev]: 5′-AGTGG CGCAATCTCGGC-3′; probe: 5′-6-FAM-AGCTACTCGGGAGGC TGAGGCAGGA-TAMRA-3′) was purchased from Integrated DNA Technologies (IDT). The signal in each sample was normalized to the amount of DNA by measuring single-copy *GAPDH* as an internal standard. To generate a standard curve, LV myocardium from a Lewis rat was mixed with either 1×10^6–1×10^1 of hAM-MSCs (n > 4) or 1,000–1 pg of hAM-MSC (n > 5). Inter-assay precision was assessed by repeating the testing of the DNA standard curve over 6 separate experiments on different days. The intermediate dilutions of the stock DNA were done independently on the day of the assay. Standard deviations were > 0.6, demonstrating high precision between qPCR assays.

Quantitative real-time PCR

To assess rat and human gene expression in the rat LV tissue, RNA was isolated using the AllPrep DNA/RNA Kit (QIAGEN) (n = 5), or RNA was isolated from cultured mouse Mφ using the RNeasy Mini Kit (n = 3–7). All RNA samples were treated with DNase I (QIAGEN). All gDNA was treated with RNase A (Thermo Fisher Scientific). cDNA was synthesized using the High-Capacity cDNA Reverse Transcription Kit (Applied Biosystems). Real-time PCR was performed using the StepOne Detection System (Applied Biosystems) with TaqMan Fast Universal PCR Master Mix (Thermo Fisher Scientific) or PowerUp SYBR Green Master Mix (Thermo Fisher Scientific) as per manufacturers' recommendations. Human TaqMan probe sets IL-10 (Hs99999035_m1), CXCL12 (Hs00930455_m1), VCAM1 (Hs01003372_m1), IGF1 (Hs00153126_m1), TGFB1 (Hs00998133_m1), HGF1 (Hs00300159_m1), MMP2 (Hs01548731_m1), IL-6 (Hs99999032_m1), IL-4 (Hs00174122_m1), GAPDH (Hs99999905_m1), rat TaqMan Il-10 (Rn00563409_m1), Cxcl12 (Rn01462853_m1), Vcam1 (Rn00563627_m1), Igf1 (Rn00710306_m1), Tgfb1 (Rn00572010_m1), tumor necrosis factor alpha (Tnfa; Rn99999017_m1), Il-6 (Rn01410330_m1), fibroblast growth factor 2 (Fgf2; Rn01462049_m1), Mrc1/Cd206 (rn01487342_m1), Cd86 (Rn00571654_m1), Cd80 (Rn00709368_m1), Timp1 (Rn00587558_m1), Mmp2 (Rn01538177_m1), Ptges2 (Rn01483828_m1), Il-4 (Rn01456866_m1), and Gapdh (Rn01775763_g1) were purchased from Thermo Fisher Scientific. Human rat and mouse SYBR Green primers were designed using Primer3 software and purchased from IDT (the full list of these primers is in Table S1). Each target gene has a minimum of three biological and three technical replicates. NTC control was used. For relative gene expression, the $2^{-\Delta\Delta CT}$ method was applied using *GAPDH* and 18S endogenous species-specific controls.

qPCR assessment of the gene transcript copies

Assessment of the number of gene transcript copies of rat and human tissue repair-related genes in xenogeneic samples containing hAM-MSCs and rat cells was carried out as previously described.[26,50,51] RNA was extracted from the LV of the hAM-MSC-dressing group

(n = 5) as described above, and the number of human or rat gene mRNA copies in 10 ng xenogeneic cDNA was calculated from their respective C_T using the linear equation from a standard curve. Standard curves were generated using a human or rat gDNA dilution series containing 1×10^7–1×10^2 gene copies calculated using the below equation.[26,27] The amplification efficiency for each primer set was determined from the linear slope of each standard curve; only primers with a standard curve slope between −3.0 and −3.9 were used for further quantification. The mass of one copy of the genes of interest was calculated from the following formula: $M = N_g \times 1.096e{-}21$ g/bp, where M = mass of the haploid genome (in grams [g]), and N_g = number of base pairs (bp) in the haploid genome. In order to avoid inter-species variation of primer-binding efficiency, we performed melting curve analysis and only used pairs of primers with similar binding efficiencies determined from a dilution curve. Rat and human GAPDH was used to normalize for loading differences.

The rat genome is estimated to be 2.87 billion bp (total sequence length as of NCBI: Rnor_6.0), whereas the human genome is estimated to be 3.09 billion bp (total sequence length as of NCBI: GRCh38.p13). Thus, one haploid rat DNA is approximately 3.14 pg, whereas one human haploid DNA is approximately 3.39 pg. qPCR was conducted with 100 ng xenogeneic LV DNA in triplicate in a 25-μL reaction using the PowerUp SYBR Green Master Mix (Thermo Fisher Scientific) and the StepOne Real-Time System (Applied Biosystems). The reaction was initiated by activation of DNA polymerase at 95°C for 2 min, followed by 40, three-step amplification cycles consisting of 3 s denaturation at 95°C and 30 s annealing/extension at 60°C. A final dissociation stage was run to generate a melting curve for verification of amplification product specificity. We ensured that the primer pair will specifically amplify the target sequence by searching for the nucleotide sequences that contain both primer sequences on opposing strands in the NCBI GenBank database using BLAST (https://www.ncbi.nlm.nih.gov/BLAST). A key consideration for using gDNA as an external standard in real-time PCR is designing target-specific primer pairs that do not span introns.

Isolation of bone marrow-derived monocytes and Mφ

Male C57BL/6 mice, 8–10 weeks old (Charles River Laboratories), were used in all experiments for the generation of monocytes and Mφ. The animals were culled by CO_2 euthanasia immediately before the start of the bone marrow isolation procedure. Mouse bone marrow was harvested as previously described.[32] Briefly bone marrow was collected by flushing the hind-limb tibia and femur with DMEM (Gibco) containing 10% FBS (Sigma-Aldrich) and 1% penicillin-streptomycin (Gibco). Cell suspension was filtered with a 70-μm cell strainer resuspended in 5 mL cold 1× red blood cell lysis buffer (BioLegend) before washing in PBS and performing gradient centrifugation using Histopaque-1077 (Sigma-Aldrich) and at 400 × *g* without brake for 30 min. The interphase was carefully collected as bone marrow mononuclear cells (BMMNCs). For monocyte collection, collected BMMNCs were subjected to magnetic sorting with the Monocyte Isolation Kit (Miltenyi Biotec) and MS Separation Columns (Miltenyi Biotec). For Mφ production, isolated BMMNCs were plated at a density of 4×10^3 cells/cm^2 and cultured for 6 days in complete DMEM containing mouse macrophage colony stimulating factor (M-CSF; 20 ng/mL; PeproTech) to differentiate to Mφ.[32]

In vitro co-culture model

Mouse Mφ were isolated as described above and seeded in 6-well plates. Medium was replaced with fresh DMEM on day 6 and then co-cultured with hAM-MSCs separated by a Transwell insert (0.4 μm pore size; Corning Life Sciences) at a ratio of 1:3 hAM-MSC:Mφ for a further 48 h (n > 10). For blocking or antibody neutralization studies, seeded hAM-MSCs were preincubated for 1 h with 30 μM COX-2 inhibitor NS-398 (Cayman Chemical) (n = 6); neutralizing antibodies from R&D Systems anti-hCXCL12 (10 μg/mL; AF-310), anti-hIL-10 (5 μg/mL; AF-217), anti-hTGF-β1 (25 μg/mL; AF-246), anti-hIGF1 (15 μg/mL; AF-291), or anti-hCCL2 (1 μg/mL; MA5-17040; Invitrogen) before co-culturing with Mφ as above (n > 4 per antibody group). Subsequently, cell culture supernatants, RNA, and whole cells were collected for enzyme-linked immunosorbent assay (ELISA), quantitative real-time PCR, and flow cytometry analysis.

Immunofluorescence staining of cultured Mφ

Glass coverslips were placed in the bottom of the wells of the culture plate and coated with 1% gelatin before cell seeding. Co-culture of Mφ and hAM-MSCs was established as described above before fixing Mφ in 4% PFA. If permeabilization were required, then cells were incubated in PBS 0.1% Triton X-100 for 10 min. Non-specific binding was blocked in PBS 1% BSA for 30 min prior to adding primary antibody. F4/80 (1:100; MA1-91124; Invitrogen), CD206 (1:150; C-20: SC-3457), or Ki67 (5 μg/mL; 14-5698; eBioscience) was added overnight at 4°C. Appropriate Alexa Flour-conjugated secondary antibodies (Thermo Fisher Scientific) were added for 1 h (1:300 dilution), and nuclei were counterstained with DAPI. Images were captured by fluorescence microscopy (BZ8000; Keyence;10×, 20×, and 40× objectives). Five fields were randomly selected per coverslip, and the average of these was taken as measurement.

Flow cytometry

Cultured and treated mouse bone-marrow-derived Mφ were detached from the culture flasks by scraping, counted, and resuspended in PBS to yield >2.5×10^5 cells/tube. Cells were resuspended in flow cytometry buffer (5% FBS, 0.002% NaN_3 in PBS) and FC receptors blocked with anti-mouse CD16/32 antibody (IgG2a, 93, monoclonal, rat; 1:100) for 30 min on ice. Conjugated antibodies CD11b-allophycocyanin (APC; 1:1,000; M1/70; Thermo Fisher Scientific), F4/80-phycoerythrin (PE; 1:400; BM-8; BioLegend), and CD206-AF488 (1:50; C082C2; BioLegend) were added for 30 min on ice.[32] IgG-conjugated-APC, PE, and Alexa Flour-488 were used as negative controls for gating purposes. Cells were resuspended in flow buffer with DAPI (2 ng/μL). Mφ surface marker expression was evaluated using the BD LSRFortessa cell analyzer, and the acquired data were further examined with FlowJo software (version [v.]10). In each sample (n > 10),

a minimum of 10,000 events were recorded. Compensation was achieved using UltraComp eBeads (Invitrogen) before each experiment. Cellular debris, doublets, and dead cells were excluded during the processing step (Figure S4).

ELISA

ELISA was performed on conditioned medium from cultured hAM-MSCs, Mφ, or cell supernatant from hAM-MSC + Mφ co-culture and analyzed using ELISA kits (Quantikine ELISA kits for mTGF-β1, mIL-10, and hPGE2 Assay; R&D Systems) according to the manufacturer's instructions (n = 3−4 per group).

Phagocytosis assay

Cultured mouse bone marrow-derived Mφ were serum starved for 2 h following 48 h hAM-MSC co-culture, 48 h IL-4 (20 ng/mL stimulation), or 18 h LPS (50 ng/mL) stimulation. Then, mIgG-coated, fluorescein isothiocyanate (FITC)-labeled beads (FluoShperes-F8803; Invitrogen) were added to cell cultures (6 particles/Mφ in DMEM containing 1% FBS) and incubated at 37°C for 45 min. Medium-only control was used as baseline. Cells were dissociated, washed twice, and resuspended in PBS containing 1% FBS. Bead uptake was quantified by flow cytometry analysis and displayed as mean fluorescence intensity (MFI) (n = 5−8).

Migration assay of monocytes and Mφ

Migration of mouse bone marrow-derived monocytes and Mφ toward the soluble factors secreted by hAM-MSCs was quantitated using 24 well Transwell inserts (6.5 mm) with polycarbonate filters (5 μm and 8 μm diameter pore size, respectively).[52,53] hAM-MSCs were seeded in a 24-well plate and grown to form a confluent monolayer before Transwells were inserted into the wells, and mouse monocytes or Mφ were added in the upper chamber of the Transwell inserts. To avoid false-positive migration toward increased serum concentrations, this assay was conducted in DMEM containing 1% FBS. Mouse cells were allowed to migrate through the insert toward hAM-MSCs for 2 h. Cells that had migrated through the Transwell membrane and adhered to the lower side of the membrane were fixed with 4% PFA, whereas non-migrated cells in the upper chamber were removed with a cotton bud. Transwell membranes containing the migrated cells were rinsed and stained with DAPI before mounting onto coverslips and counting stained cells using a fluorescence microscope (10× objective). To assess the role of hAM-MSC-secreted CCL2 on monocyte and Mφ migration, a neutralizing antibody was used (1 μg/mL) along with a CCL2 recombinant protein control (50 ng/mL). Conditions were repeated in duplicate for each bone marrow isolation, and an average >5 frames per insert were captured (n = 5 for the monocyte study; n = 4 for the Mφ study).

Statistical analysis

The statistical analysis was conducted using GraphPad Prism (v.8) software. Methods of statistical analysis used are stated in each figure legend. The significance threshold was set at $p < 0.05$.

SUPPLEMENTAL INFORMATION

Supplemental information can be found online at https://doi.org/10.1016/j.ymthe.2021.04.018.

ACKNOWLEDGMENTS

All animal studies were performed with the approval of the Institutional Ethics Committee at Queen Mary University of London and the UK Home Office. The investigation conforms to the Principles of Laboratory Animal Care formulated by the National Society for Medical Research and the *Guide for the Care and Use of Laboratory Animals* (US NIH Publication, 1996). We thank Dr. Kenichi Yamahara (Hyogo Medical College, Japan) for a gift of hAM-MSCs. We also appreciate technical assistance of Dr. Maryam Jangani and Dr. Hidetoshi Hoshiya. This project was funded by the British Heart Foundation (program grants RG/15/3/31236 and RG/19/7/34577 and project grant PG/18/77/34100). It was also supported by the UK National Institute for Health Research Biomedical Research Centre at Barts.

AUTHOR CONTRIBUTIONS

Conceptualization, L.F. and K.S.; methodology, L.F., T.I., and K.S.; funding acquisition, K.S.; investigation, L.F., K.K., Y.I., M.-N.P., M.H., T.I., K.Y., and V.M.; analysis, L.F.; visualization, L.F.; supervision, K.S.; writing – original draft, L.F. and K.S.; writing – review & editing, F.L.-M. and K.S.

DECLARATION OF INTERESTS

The authors declare no competing interests.

REFERENCES

1. Virani, S.S., Alonso, A., Benjamin, E.J., Bittencourt, M.S., Callaway, C.W., Carson, A.P., Chamberlain, A.M., Chang, A.R., Cheng, S., Delling, F.N., et al.; American Heart Association Council on Epidemiology and Prevention Statistics Committee and Stroke Statistics Subcommittee (2020). Heart Disease and Stroke Statistics-2020 Update: A Report From the American Heart Association. Circulation *141*, e139–e596.
2. Kobayashi, K., and Suzuki, K. (2018). Mesenchymal Stem/Stromal Cell-Based Therapy for Heart Failure - What Is the Best Source? Circ. J. *82*, 2222–2232.
3. van der Spoel, T.I.G., Jansen of Lorkeers, S.J., Agostoni, P., van Belle, E., Gyöngyösi, M., Sluijter, J.P.G., Cramer, M.J., Doevendans, P.A., and Chamuleau, S.A.J. (2011). Human relevance of pre-clinical studies in stem cell therapy: systematic review and meta-analysis of large animal models of ischaemic heart disease. Cardiovasc. Res. *91*, 649–658.
4. Narita, T., and Suzuki, K. (2015). Bone marrow-derived mesenchymal stem cells for the treatment of heart failure. Heart Fail. Rev. *20*, 53–68.
5. Hare, J.M., Traverse, J.H., Henry, T.D., Dib, N., Strumpf, R.K., Schulman, S.P., Gerstenblith, G., DeMaria, A.N., Denktas, A.E., Gammon, R.S., et al. (2009). A randomized, double-blind, placebo-controlled, dose-escalation study of intravenous adult human mesenchymal stem cells (prochymal) after acute myocardial infarction. J. Am. Coll. Cardiol. *54*, 2277–2286.
6. Jeong, H., Yim, H.W., Park, H.J., Cho, Y., Hong, H., Kim, N.J., and Oh, I.H. (2018). Mesenchymal Stem Cell Therapy for Ischemic Heart Disease: Systematic Review and Meta-analysis. Int. J. Stem Cells *11*, 1–12.
7. Fukushima, S., Sawa, Y., and Suzuki, K. (2013). Choice of cell-delivery route for successful cell transplantation therapy for the heart. Future Cardiol. *9*, 215–227.
8. Campbell, N.G., Kaneko, M., Shintani, Y., Narita, T., Sawhney, V., Coppen, S.R., Yashiro, K., Mathur, A., and Suzuki, K. (2016). Cell Size Critically Determines

Initial Retention of Bone Marrow Mononuclear Cells in the Heart after Intracoronary Injection: Evidence from a Rat Model. PLoS ONE *11*, e0158232.

9. Kobayashi, K., Ichihara, Y., Sato, N., Umeda, N., Fields, L., Fukumitsu, M., Tago, Y., Ito, T., Kainuma, S., Podaru, M., et al. (2019). On-site fabrication of Bi-layered adhesive mesenchymal stromal cell-dressings for the treatment of heart failure. Biomaterials *209*, 41–53.

10. Tsuji, H., Miyoshi, S., Ikegami, Y., Hida, N., Asada, H., Togashi, I., Suzuki, J., Satake, M., Nakamizo, H., Tanaka, M., et al. (2010). Xenografted human amniotic membrane-derived mesenchymal stem cells are immunologically tolerated and transdifferentiated into cardiomyocytes. Circ. Res. *106*, 1613–1623.

11. Lindenmair, A., Hatlapatka, T., Kollwig, G., Hennerbichler, S., Gabriel, C., Wolbank, S., Redl, H., and Kasper, C. (2012). Mesenchymal stem or stromal cells from amnion and umbilical cord tissue and their potential for clinical applications. Cells *1*, 1061–1088.

12. Gnecchi, M., Danieli, P., Malpasso, G., and Ciuffreda, M.C. (2016). Paracrine Mechanisms of Mesenchymal Stem Cells in Tissue Repair. Methods Mol. Biol. *1416*, 123–146.

13. Hodgkinson, C.P., Bareja, A., Gomez, J.A., and Dzau, V.J. (2016). Emerging Concepts in Paracrine Mechanisms in Regenerative Cardiovascular Medicine and Biology. Circ. Res. *118*, 95–107.

14. Németh, K., Leelahavanichkul, A., Yuen, P.S.T., Mayer, B., Parmelee, A., Doi, K., Robey, P.G., Leelahavanichkul, K., Koller, B.H., Brown, J.M., et al. (2009). Bone marrow stromal cells attenuate sepsis via prostaglandin E(2)-dependent reprogramming of host macrophages to increase their interleukin-10 production. Nat. Med. *15*, 42–49.

15. Kim, J., and Hematti, P. (2009). Mesenchymal stem cell-educated macrophages: a novel type of alternatively activated macrophages. Exp. Hematol. *37*, 1445–1453.

16. Zhang, B., Zhao, N., Zhang, J., Liu, Y., Zhu, D., and Kong, Y. (2019). Mesenchymal stem cells rejuvenate cardiac muscle through regulating macrophage polarization. Aging (Albany NY) *11*, 3900–3908.

17. Wagner, M.J., Khan, M., and Mohsin, S. (2020). Healing the Broken Heart; The Immunomodulatory Effects of Stem Cell Therapy. Front. Immunol. *11*, 639.

18. Ben-Mordechai, T., Holbova, R., Landa-Rouben, N., Harel-Adar, T., Feinberg, M.S., Abd Elrahman, I., Blum, G., Epstein, F.H., Silman, Z., Cohen, S., and Leor, J. (2013). Macrophage subpopulations are essential for infarct repair with and without stem cell therapy. J. Am. Coll. Cardiol. *62*, 1890–1901.

19. Jin, L., Deng, Z., Zhang, J., Yang, C., Liu, J., Han, W., Ye, P., Si, Y., and Chen, G. (2019). Mesenchymal stem cells promote type 2 macrophage polarization to ameliorate the myocardial injury caused by diabetic cardiomyopathy. J. Transl. Med. *17*, 251.

20. Dayan, V., Yannarelli, G., Billia, F., Filomeno, P., Wang, X.H., Davies, J.E., and Keating, A. (2011). Mesenchymal stromal cells mediate a switch to alternatively activated monocytes/macrophages after acute myocardial infarction. Basic Res. Cardiol. *106*, 1299–1310.

21. Liu, F., Qiu, H., Xue, M., Zhang, S., Zhang, X., Xu, J., Chen, J., Yang, Y., and Xie, J. (2019). MSC-secreted TGF-β regulates lipopolysaccharide-stimulated macrophage M2-like polarization via the Akt/FoxO1 pathway. Stem Cell Res. Ther. *10*, 345.

22. Vasandan, A.B., Jahnavi, S., Shashank, C., Prasad, P., Kumar, A., and Prasanna, S.J. (2016). Human Mesenchymal stem cells program macrophage plasticity by altering their metabolic status via a PGE_2-dependent mechanism. Sci. Rep. *6*, 38308.

23. Giri, J., Das, R., Nylen, E., Chinnadurai, R., and Galipeau, J. (2020). CCL2 and CXCL12 Derived from Mesenchymal Stromal Cells Cooperatively Polarize IL-10+ Tissue Macrophages to Mitigate Gut Injury. Cell Rep. *30*, 1923–1934.e4.

24. Zhao, J., Li, X., Hu, J., Chen, F., Qiao, S., Sun, X., Gao, L., Xie, J., and Xu, B. (2019). Mesenchymal stromal cell-derived exosomes attenuate myocardial ischaemia-reperfusion injury through miR-182-regulated macrophage polarization. Cardiovasc. Res. *115*, 1205–1216.

25. Burchfield, J.S., Xie, M., and Hill, J.A. (2013). Pathological ventricular remodeling: mechanisms: part 1 of 2. Circulation *128*, 388–400.

26. Yun, J.J., Heisler, L.E., Hwang, I.I.L., Wilkins, O., Lau, S.K., Hyrcza, M., Jayabalasingham, B., Jin, J., McLaurin, J., Tsao, M.S., and Der, S.D. (2006). Genomic DNA functions as a universal external standard in quantitative real-time PCR. Nucleic Acids Res. *34*, e85.

27. Applied Biosystems (2003). Creating Standard Curves with Genomic DNA or Plasmid DNA Templates for Use in Quantitative PCR, http://tools.thermofisher.com/content/sfs/brochures/cms_042486.pdf.

28. Lee, C., Kim, J., Shin, S.G., and Hwang, S. (2006). Absolute and relative QPCR quantification of plasmid copy number in Escherichia coli. J. Biotechnol. *123*, 273–280.

29. Rőszer, T. (2015). Understanding the Mysterious M2 Macrophage through Activation Markers and Effector Mechanisms. Mediators Inflamm. *2015*, 816460.

30. Gordon, S. (2003). Alternative activation of macrophages. Nat. Rev. Immunol. *3*, 23–35.

31. Balce, D.R., Li, B., Allan, E.R.O., Rybicka, J.M., Krohn, R.M., and Yates, R.M. (2011). Alternative activation of macrophages by IL-4 enhances the proteolytic capacity of their phagosomes through synergistic mechanisms. Blood *118*, 4199–4208.

32. Podaru, M.N., Fields, L., Kainuma, S., Ichihara, Y., Hussain, M., Ito, T., Kobayashi, K., Mathur, A., D'Acquisto, F., Lewis-McDougall, F., and Suzuki, K. (2019). Reparative macrophage transplantation for myocardial repair: a refinement of bone marrow mononuclear cell-based therapy. Basic Res. Cardiol. *114*, 34.

33. Gong, D., Shi, W., Yi, S.J., Chen, H., Groffen, J., and Heisterkamp, N. (2012). TGFβ signaling plays a critical role in promoting alternative macrophage activation. BMC Immunol. *13*, 31.

34. Futaki, N., Takahashi, S., Yokoyama, M., Arai, I., Higuchi, S., and Otomo, S. (1994). NS-398, a new anti-inflammatory agent, selectively inhibits prostaglandin G/H synthase/cyclooxygenase (COX-2) activity in vitro. Prostaglandins *47*, 55–59.

35. Deshmane, S.L., Kremlev, S., Amini, S., and Sawaya, B.E. (2009). Monocyte chemoattractant protein-1 (MCP-1): an overview. J. Interferon Cytokine Res. *29*, 313–326.

36. Nesteruk, J., Voronina, N., Kundt, G., Donndorf, P., Klopsch, C., Kaminski, A., Duckers, H.J., and Steinhoff, G. (2017). Stem cell registry programme for patients with ischemic cardiomyopathy undergoing coronary artery bypass grafting: what benefits does it derive? ESC Heart Fail. *4*, 105–111.

37. Lee, T. (2010). Host tissue response in stem cell therapy. World J. Stem Cells *2*, 61–66.

38. Shiraishi, M., Shintani, Y., Shintani, Y., Ishida, H., Saba, R., Yamaguchi, A., Adachi, H., Yashiro, K., and Suzuki, K. (2016). Alternatively activated macrophages determine repair of the infarcted adult murine heart. J. Clin. Invest. *126*, 2151–2166.

39. Shintani, Y., Ito, T., Fields, L., Shiraishi, M., Ichihara, Y., Sato, N., Podaru, M., Kainuma, S., Tanaka, H., and Suzuki, K. (2017). IL-4 as a Repurposed Biological Drug for Myocardial Infarction through Augmentation of Reparative Cardiac Macrophages: Proof-of-Concept Data in Mice. Sci. Rep. *7*, 6877.

40. Papa, S., Vismara, I., Mariani, A., Barilani, M., Rimondo, S., De Paola, M., Panini, N., Erba, E., Mauri, E., Rossi, F., et al. (2018). Mesenchymal stem cells encapsulated into biomimetic hydrogel scaffold gradually release CCL2 chemokine in situ preserving cytoarchitecture and promoting functional recovery in spinal cord injury. J. Control. Release *278*, 49–56.

41. Takeda, K., Webb, T.L., Ning, F., Shiraishi, Y., Regan, D.P., Chow, L., Smith, M.J., Ashino, S., Guth, A.M., Hopkins, S., et al. (2018). Mesenchymal Stem Cells Recruit $CCR2^+$ Monocytes To Suppress Allergic Airway Inflammation. J. Immunol. *200*, 1261–1269.

42. Ohshima, M., Yamahara, K., Ishikane, S., Harada, K., Tsuda, H., Otani, K., Taguchi, A., Miyazato, M., Katsuragi, S., Yoshimatsu, J., et al. (2012). Systemic transplantation of allogenic fetal membrane-derived mesenchymal stem cells suppresses Th1 and Th17 T cell responses in experimental autoimmune myocarditis. J. Mol. Cell. Cardiol. *53*, 420–428.

43. Cho, H.J., Lee, N., Lee, J.Y., Choi, Y.J., Ii, M., Wecker, A., Jeong, J.O., Curry, C., Qin, G., and Yoon, Y.S. (2007). Role of host tissues for sustained humoral effects after endothelial progenitor cell transplantation into the ischemic heart. J. Exp. Med. *204*, 3257–3269.

44. Kamata, S., Miyagawa, S., Fukushima, S., Nakatani, S., Kawamoto, A., Saito, A., Harada, A., Shimizu, T., Daimon, T., Okano, T., et al. (2014). Improvement of cardiac stem cell sheet therapy for chronic ischemic injury by adding endothelial progenitor cell transplantation: analysis of layer-specific regional cardiac function. Cell Transplant. *23*, 1305–1319.

45. Kubo, K., Ohnishi, S., Hosono, H., Fukai, M., Kameya, A., Higashi, R., Yamada, T., Onishi, R., Yamahara, K., Takeda, H., and Sakamoto, N. (2015). Human Amnion-

Derived Mesenchymal Stem Cell Transplantation Ameliorates Liver Fibrosis in Rats. Transplant. Direct *1*, e16.

46. Kobayashi, K., Ichihara, Y., Tano, N., Fields, L., Murugesu, N., Ito, T., Ikebe, C., Lewis, F., Yashiro, K., Shintani, Y., et al. (2018). Fibrin Glue-aided, Instant Epicardial Placement Enhances the Efficacy of Mesenchymal Stromal Cell-Based Therapy for Heart Failure. Sci. Rep. *8*, 9448.

47. Ichihara, Y., Kaneko, M., Yamahara, K., Koulouroudias, M., Sato, N., Uppal, R., Yamazaki, K., Saito, S., and Suzuki, K. (2018). Self-assembling peptide hydrogel enables instant epicardial coating of the heart with mesenchymal stromal cells for the treatment of heart failure. Biomaterials *154*, 12–23.

48. Tano, N., Kaneko, M., Ichihara, Y., Ikebe, C., Coppen, S.R., Shiraishi, M., Shintani, Y., Yashiro, K., Warrens, A., and Suzuki, K. (2016). Allogeneic Mesenchymal Stromal Cells Transplanted Onto the Heart Surface Achieve Therapeutic Myocardial Repair Despite Immunologic Responses in Rats. J. Am. Heart Assoc. *5*, e002815.

49. Francis, J., Weiss, R.M., Wei, S.G., Johnson, A.K., and Felder, R.B. (2001). Progression of heart failure after myocardial infarction in the rat. Am. J. Physiol. Regul. Integr. Comp. Physiol. *281*, R1734–R1745.

50. Prigent, J., Herrero, A., Ambroise, J., Smets, F., Deblandre, G.A., and Sokal, E.M. (2015). Human Progenitor Cell Quantification After Xenotransplantation in Rat and Mouse Models by a Sensitive qPCR Assay. Cell Transplant. *24*, 1639–1652.

51. Alcoser, S.Y., Kimmel, D.J., Borgel, S.D., Carter, J.P., Dougherty, K.M., and Hollingshead, M.G. (2011). Real-time PCR-based assay to quantify the relative amount of human and mouse tissue present in tumor xenografts. BMC Biotechnol. *11*, 124.

52. Hu, Y., Hu, X., Boumsell, L., and Ivashkiv, L.B. (2008). IFN-gamma and STAT1 arrest monocyte migration and modulate RAC/CDC42 pathways. J. Immunol. *180*, 8057–8065.

53. Green, T.D., Park, J., Yin, Q., Fang, S., Crews, A.L., Jones, S.L., and Adler, K.B. (2012). Directed migration of mouse macrophages in vitro involves myristoylated alanine-rich C-kinase substrate (MARCKS) protein. J. Leukoc. Biol. *92*, 633–639.

YMTHE, Volume *29*

Supplemental Information

Epicardial placement of human MSC-loaded fibrin sealant films for heart failure: Preclinical efficacy and mechanistic data

Laura Fields, Tomoya Ito, Kazuya Kobayashi, Yuki Ichihara, Mihai-Nicolae Podaru, Mohsin Hussain, Kizuku Yamashita, Vanessa Machado, Fiona Lewis-McDougall, and Ken Suzuki

SUPPLEMENTAL INFORMATION

Epicardial Placement of Fibrin Sealant Film-incorporating Human Mesenchymal Stromal Cells for the Treatment of Heart Failure: Towards Clinical Translation and Mechanistic Implication

Short Title: Fields et al. MSC-Dressing Therapy

Supplemental Information includes:

- Supplemental Figures S1-S8 with legends
- Supplemental Table S1

Figure S1

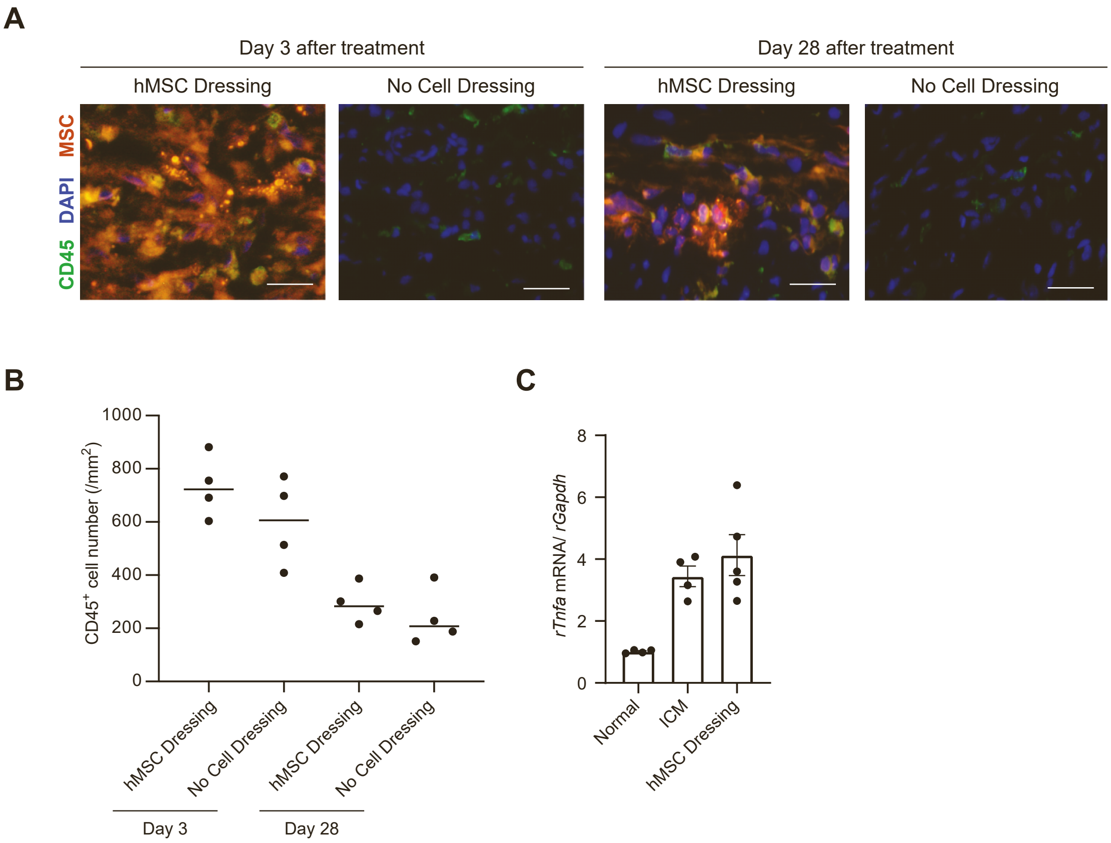

Figure S1. Immunological response induced by hAM-MSC-dressing therapy.

Four weeks after left coronary artery ligation in rats, a hAM-MSC-dressing (a fibrin sealant film containing 2 $\times 10^6$ hAM-MSCs; hMSC-dressing group), a fibrin sealant film only (No Cell-dressing group), or nothing (no treatment; ICM group) were placed onto the rat heart surface.

(A) Representative images of immunohistolabeling for CD45 (green) with (DAPI; blue) staining exhibiting the $CD45^+$ cell accumulation in the construct of hAM-MSC-dressing or No Cell-dressing at day 3 and 28 after treatment. Scale bars = 20 µm. n = 4 rats in each group.

(B) The graphs presenting the number of $CD45^+$ cells in the construct of hAM-MSC-dressing or No Cell-dressing at day 3 and 28 after treatment. n = 4 rats in each group.

(C) RT-qPCR analysis data showing increased expression of *rTnfa* genes of the rat LV samples of the hAM-MSC group, as compared to the ICM group, at day 3 post treatment. Normal (no MI, no treatment) group was included as a reference. n = 5 hearts in each group. Data are presented as mean ± SEM, Student's t-test for statistical analysis of (B); One-way ANOVA with Tukey's post-hoc tests for (C).

Figure S2

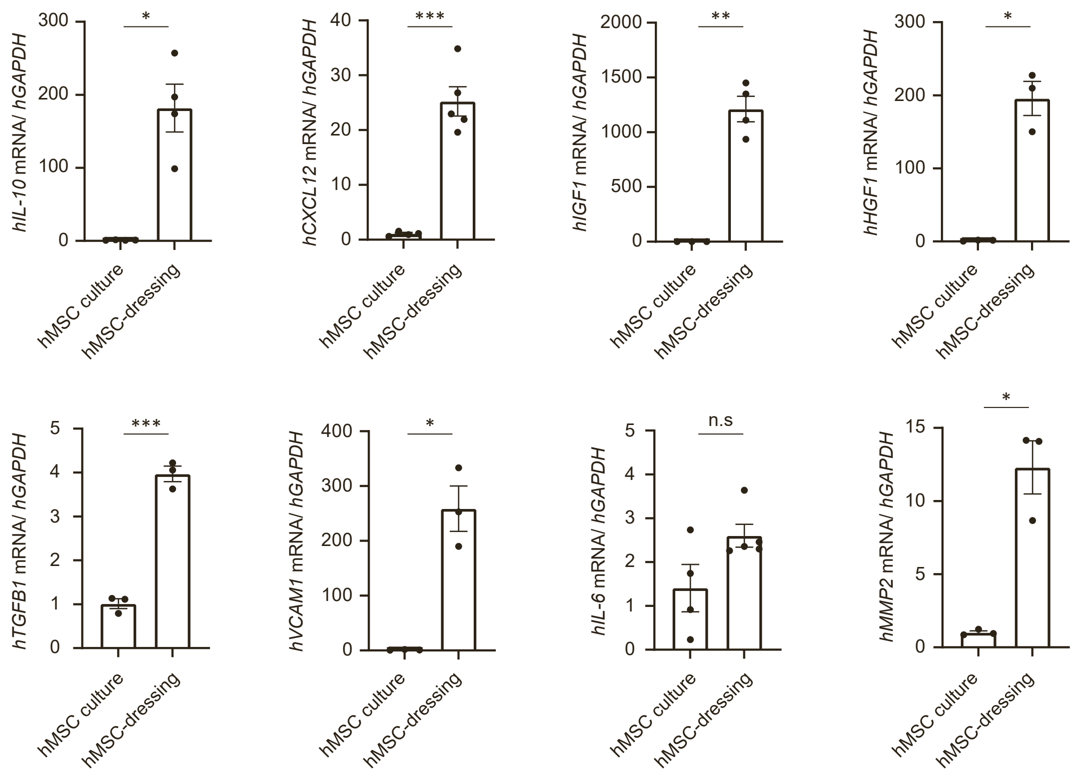

Figure S2. Expression of human reparative genes in the rat heart post hAM-MSC-dressing therapy.

Four weeks after left coronary artery ligation in rats, hAM-MSC-dressings (fibrin sealant film containing 2×10^6 hAM-MSCs; hMSC-dressing group) were placed onto the heart surface. At day 3 post-transplantation, RNA was extracted from the rat LV tissue for RT-qPCR for tissue repair-related human genes. As a control, RNA was extracted from ordinarily cultured hAM-MSCs was used. n = 5 in each group. Data are presented as mean ± SEM, *p < 0.05, **p < 0.01, ***p < 0.001, n.s = not significant. Student’s t-test.

Figure S3

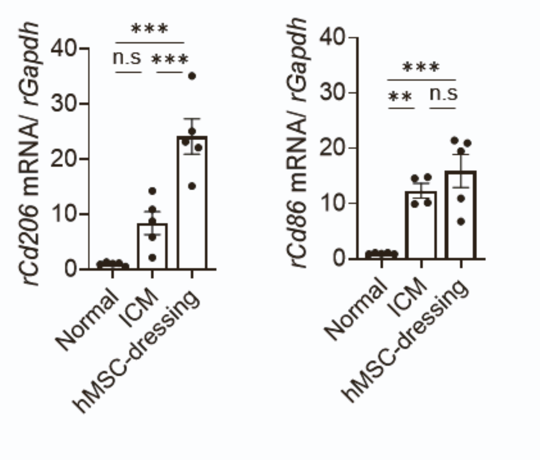

Figure S3. Expression of Mφ phenotype markers in the rat heart post hAM-MSC-dressing therapy.

Rat hearts were collected at Day 3 after hAM-MSC-dressing therapy or no treatment (ICM group) in a rat ICM model and subjected to RT-qPCR for *Cd206* and *Cd86*. Normal (no MI, no treatment) group was included as a reference. n = 5 hearts in each group. Data are presented as mean ± SEM, $^{**}p < 0.01$, $^{***}p < 0.001$, n.s = not significant. One-way ANOVA with Tukey's post-hoc test.

Figure S4

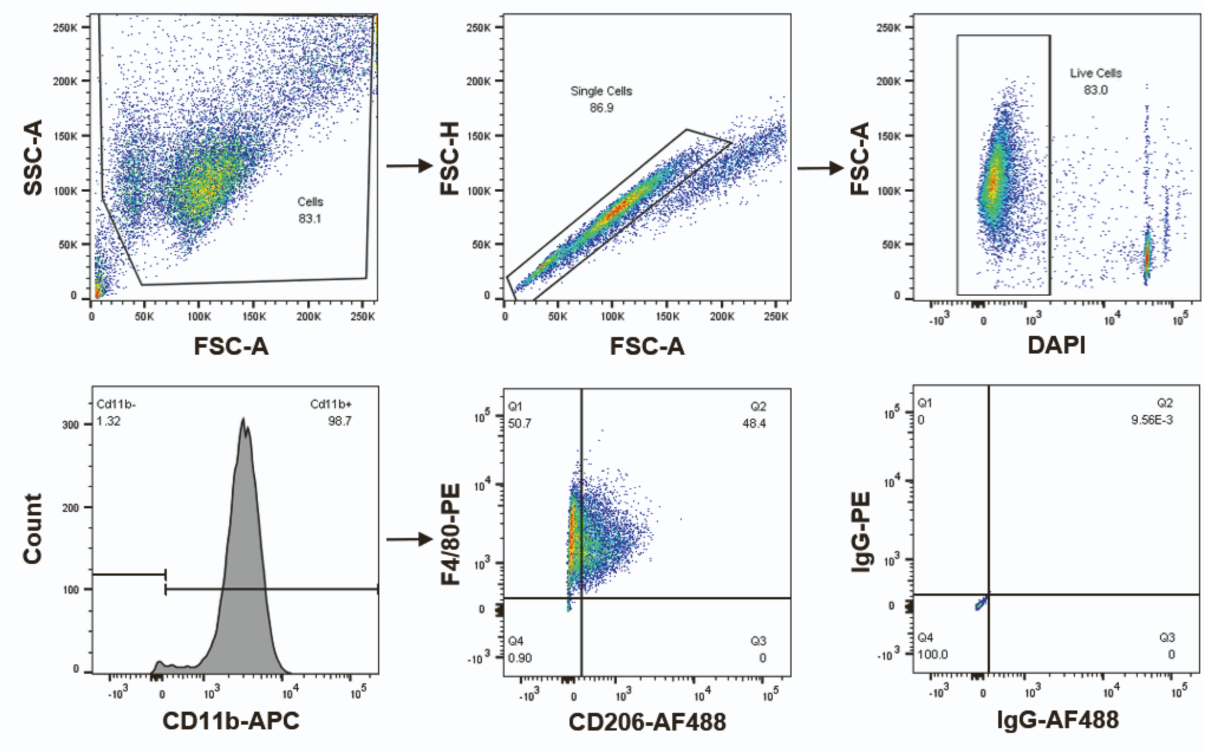

Figure S4. Flow cytometry gating to characterize M2Mφ.

Gating strategy to detect M2Mφ by flow cytometry is illustrated. Debris, doublets and dead cells are excluded. Cells double positive for F4/80 and CD206 within the $CD11b^+$ population were defined as M2Mφ by flow cytometry. Representative dot plots from a sample of M-CSF + hAM-MSC treated mouse BM-MNCs are presented. The same cells stained with IgG conjugated fluorescent antibodies shown as negative control. FMO control also used.

Figure S5

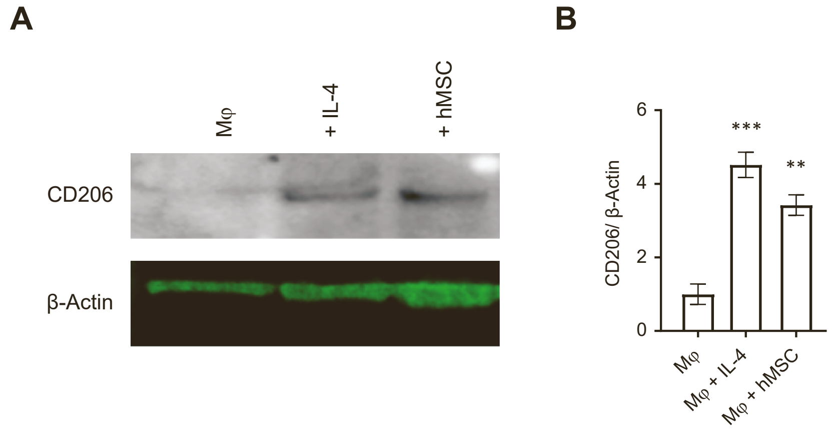

Figure S5. Enhanced CD206 protein expression in Mφ by coculture with hAM-MSCs.

Mouse bone marrow-derived Mφ were cultured alone, with IL-4 (20ng/ ml) or hAM-MSCs in a non-contact co-culture model for 48 hours. Cells were collected and CD206 protein expression levels were analyzed by western blot technique.

(A) Representative blot showing higher CD206 expression in Mφ after IL-4 and hAM-MSC treatment compared to unstimulated Mφ. β-Actin was used as loading control.

(B) Quantification of CD206 expression. n = 3 animals. Data are presented as mean fold change ± SEM compared to Mφ, $^{**}p < 0.01$, $^{***}p < 0.001$, One-way ANOVA with Dunnett's post-hoc test.

Figure S6

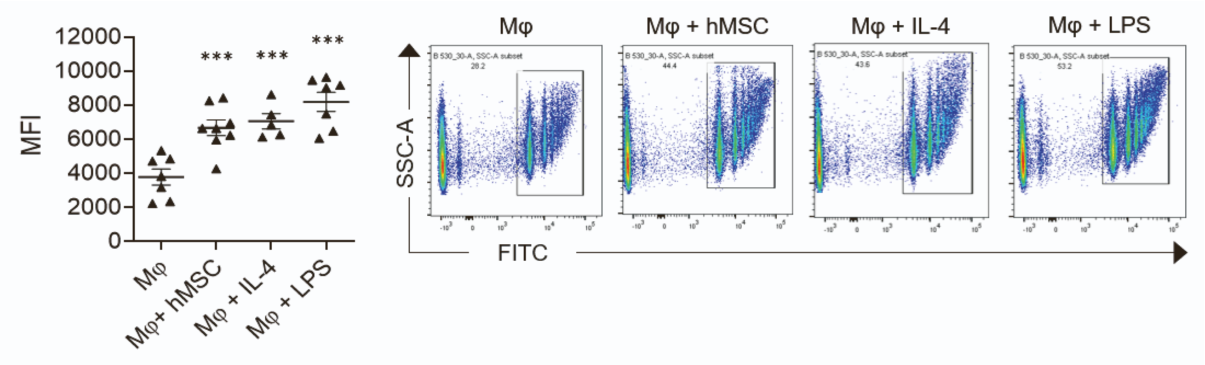

Figure S6. Increased Mφ phagocytotic activity by co-culture with hAM-MSCs.

Mouse bone marrow-derived Mφ were subjected to hAM-MSC transwell co-culture conditions before adding FITC-labelled beads in serum free media. Positive controls of LPS (50ng/ ml) or IL-4 (20ng/ ml) administration was included as well as an unstimulated (MCSF only) Mφ treatment group. Cells containing the FITC^{+} beads were assessed by flow cytometry. The mean fluorescence intensity (MFI) was quantified (left graph). Representative flow cytometry images for each group are shown in the panel on the right. n = 5-7. Data are presented as mean ± SEM, ***$p < 0.001$ compared to Mφ, One-way ANOVA with Dunnett's post-hoc test.

Figure S7

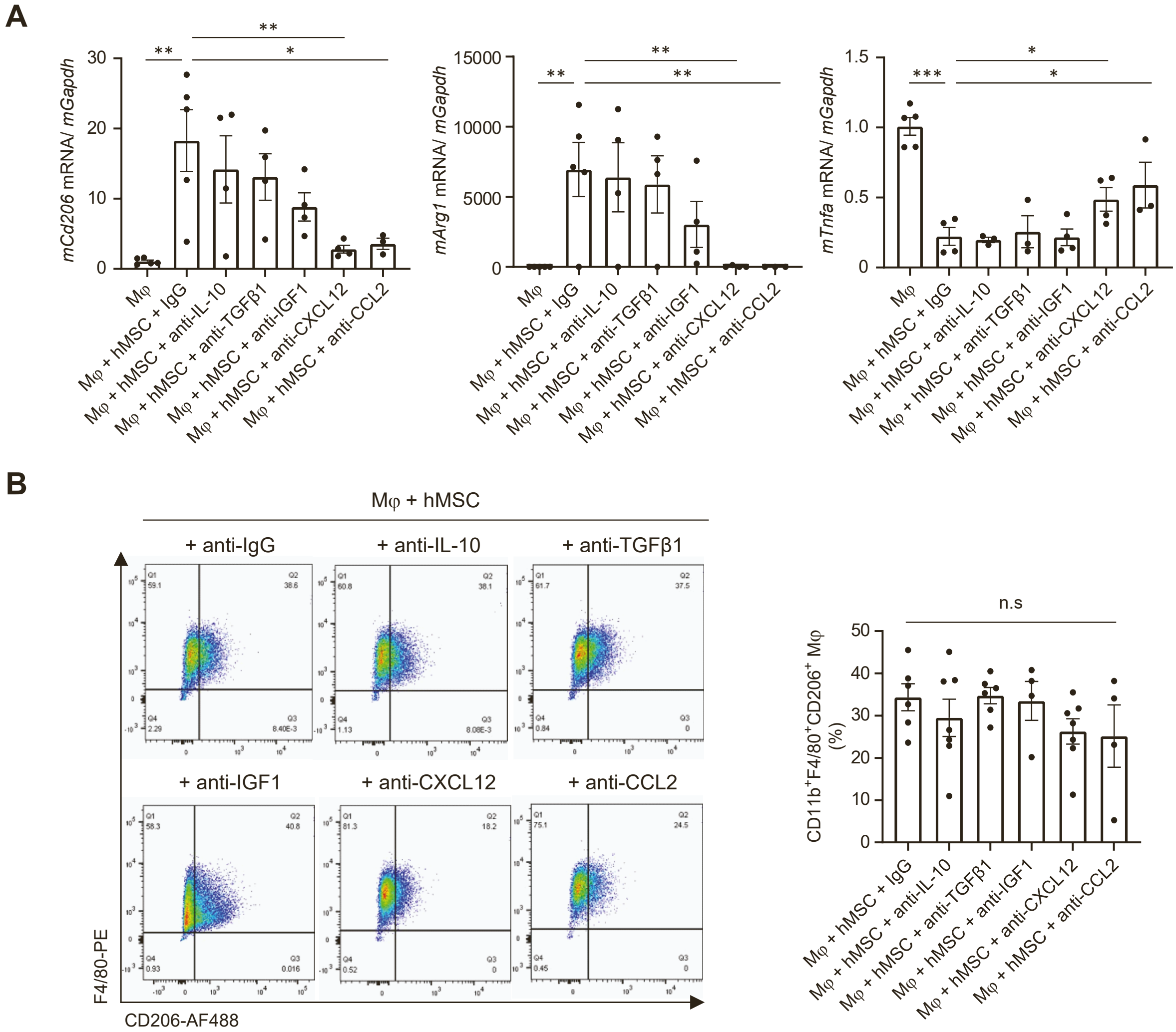

Figure S7. Role of CXCL12 and CCL2 in hAM-MSC-induced M2Mφ polarization.

Mouse bone marrow-derived Mφ were mono-cultured (Mφ group) or co-cultured with hAM-MSCs in the presence of neutralizing antibodies for human IL-10 (Mφ + hMSC + anti-IL-10), TGFβ1 (Mφ + hMSC + anti-TGFβ1), IGF1 (Mφ + hMSC + anti-IGF1), CXCL12 (Mφ + hMSC + anti-CXCL12) and CCL2 (Mφ + hMSC + anti-CCL2) or IgG antibody control (Mφ + hMSC + IgG). After 48 hours Mφ were collected and assessed for M2Mφ production.

(A) Gene expression of Mφ in each group measured by RT-qPCR. *Gapdh* was used as a reference gene. n = 4-5 biological replicates with 3 technical replicates.

(B) The percentage of M2Mφ ($CD11b^+F4/80^+CD206^+$) to the total live cell population quantified by flow cytometry. Representative dot plots and a bar chart summarizing the data are presented. n = 4-8 in each group.

Data are presented as mean ± SEM. *$p < 0.05$, **$p < 0.01$, ***$p < 0.001$. One-way ANOVA with Dunnett's multiple comparisons test.

Figure S8

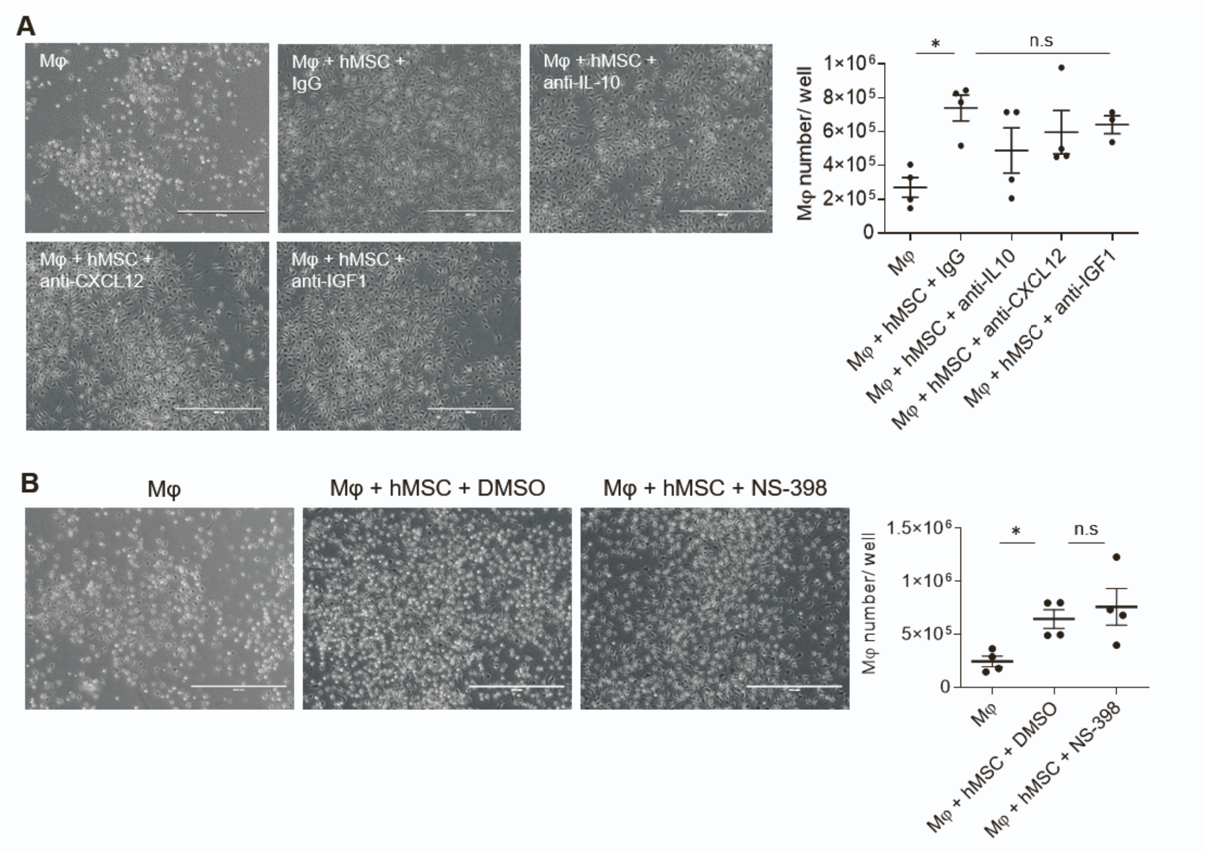

Figure S8. Inhibition of hAM-MSC-secreted PGE2, IL-10, CXCL12 or IGF1 did not affect Mφ proliferation.

(A) Mouse bone marrow-derived Mφ were co-cultured for 48 with hAM-MSCs in the presence of neutralizing antibodies for human IL-10 (Mφ + hMSC + anti-IL-10), IGF1 (Mφ + hMSC + anti-IGF1), CXCL12 (Mφ + hMSC + anti-CXCL12) or IgG antibody controls (Mφ + hMSC + IgG). Cells were collected and the numbers were quantified. Representative phase contrast images and a chart showing the cell number counted are presented. Scale bar = 400 µm. n=3-4 per group.

(B) Mouse bone marrow-derived Mφ were co-cultured for 48 with hAM-MSCs pre-treated with DMSO (vehicle control; Mφ + hMSC + DMSO group) or COX-2 inhibitor, NS-398, in DEMSO (Mφ + hMSC + NS-398 group). Representative phase contrast images and a chart showing the cell number counted are presented. Scale bar = 400 µm. n = 4 in each group.

Data are presented as mean ± SEM. *$p < 0.05$; n.s = not significant. One-way ANOVA with Dunnett's comparisons test.

Table S1. Primer sequences

Gene	Primer Sequence
mArg1	Forward:5'-CAAGACAGGGCTCCTTTCAG-3'<br>Reverse: 5'-AAGCAAGCCAAGGTTAAAGC-3'
mFizz1	Forward:5'-AGGAACTTCTTGCCAATCCA-3'<br>Reverse: 5'-ACAAGCACACCCAGTAGCAG-3'
mIl-10	Forward:5'-GGACAACATACTGCTAAAGGACTCCT-3'<br>Reverse: 5'-GCCTGGGGCATCACTTCTAC-3'
mTgfb1	Forward:5'-CCTATATTTGGAGCCTGGACACAC-3'<br>Reverse: 5'-GCTTGCGACCCACGTAGTAGA-3'
mIgf1	Forward:5'-GGACCGAGGGGCTTTTACTTC-3'<br>Reverse: 5'-GGCACAGTACATCTCCAGTCTCCTC-3'
mTnfa	Forward:5'-ATGGCCTCCCTCTGATCAGTT-3'<br>Reverse: 5'-TCTTTGAGATCCATGCCGTTG-3'
mCd206	Forward:5'-ACTACACACTCATCCATTACAACCAA-3'<br>Reverse: 5'-GGCACCTATCACAATCAGGAGGA-3'
mInos	Forward:5'- CTCCATGACTCCCAGCACAAA-3'<br>Reverse: 5'- CACTCTCTTGCGGACCATCTC-3'
mIl-1b	Forward:5'- CAAGCAACGACAAAATACCTGTG-3'<br>Reverse: 5'- ACCGTTTTTCCATCTTCTTCTTTGG-3'
mCd86	Forward:5'- GTTACTGTGGCCCTCCTCCTTGT-3'<br>Reverse: 5'- TGTCAGCGTTACTATCCCGCTCT-3'
rCxcl12	Forward:5'- CTGGATAATGTGAGAACATGCCTAGAA-3'<br>Reverse: 5'- TGCAAAAGTCCAATTCCTCAAA-3'
rVcam1	Forward:5'- CTGAGTGCAAGAAGCCAACTAGAAA-3'<br>Reverse: 5'- CTACTACTCTAAACGACCTCGCAATGA-3'
rIl-10	Forward:5'- CACCCGGCATCTACTGGACT-3'<br>Reverse: 5'- TATTTTGGAGAGAGGTACAAACGAGGT-3'
rIl-6	Forward:5'- GCCTAAGCATATCAGTTTGTGGACATT-3'<br>Reverse: 5'- AACATTCATATTGCCAGTTCTTCGT-3'
rTgfb1	Forward:5'- GACTCTCCACCTGCAAGACCATC-3'<br>Reverse: 5'- GGACTGGCGAGCCTTAGTTTG-3'

rIl-4	Forward:5'- CTGTAGAGAGCTATTGATGGGTCTCAG-3'<br>Reverse: 5'- CTTTTTCTGTGACCTGGTTCAAAGTG-3'
rIgf1	Forward:5'- AAACAAATAGAATAACAATAACTATGACTTTGAGG-3'<br>Reverse: 5'- TTGAAGGAACCATTGAGAGTTTAAGAG-3'
hCXCL12	Forward:5'- CTTGTAATCCGAATCTCTTTTTGCTTT-3'<br>Reverse: 5'- GCCCAAGGGAGTGTCAGGTAG-3'
hVCAM1	Forward:5'- TACTGCTCATCATTCCTTGAGAAAAAC-3'<br>Reverse: 5'- GCTCACAGCAAGGGACATAGA-3'
hIL-10	Forward:5'- ACGCTTTCTAGCTGTTGAGCTGTTTT-3'<br>Reverse: 5'- GGCTCCCTGGTTTCTCTTCCT-3'
hIL-6	Forward:5'- ATGGGCACCTCAGATTGTTGTT-3'<br>Reverse: 5'- GTGTCCTAACGCTCATACTTTTAGTTC-3'
hTGFB1	Forward:5'- CTCATTCAGTCACCATAGCAACACTC-3'<br>Reverse: 5'- CTTCTTCACTATCCCCCACTAAAGCA-3'
hIL-4	Forward:5'- ACTGCTTCCCCCTCTGTTCT-3'<br>Reverse: 5'- CTTCTGCTCTGTGAGGCTGTT-3'
hIGF1	Forward:5'- ACCACCTTTCAACTTTTTATCACTCAC-3'<br>Reverse: 5'- CAACAAAACAATGGAGCCTTCTAAC-3'
